# Chlorin e6-associated photodynamic therapy enhances abscopal antitumor effects via inhibition of PD-1/PD-L1 immune checkpoint

**DOI:** 10.1038/s41598-023-30256-0

**Published:** 2023-03-21

**Authors:** Pallavi Gurung, Junmo Lim, Rajeev Shrestha, Yong-Wan Kim

**Affiliations:** Dongsung Cancer Center, Dongsung Biopharmaceutical, Daegu, 41061 South Korea

**Keywords:** Biochemistry, Cancer

## Abstract

We hypothesized that photodynamic therapy (PDT) with Chlorin e6 (Ce6) enhances antitumor abscopal effects via inhibition of the programmed cell death-1/programmed death-ligand 1 (PD-1/PD-L1) immune checkpoint. By using syngeneic melanoma and pancreatic tumor mouse models, we studied the Ce6-PDT-induced immune responses in local and distant tumor microenvironments. In addition, the Ce6-PDT's target in the PD-1/PD-L1 interaction was analyzed in MC38-hPD-L1 colon cancer and PD-1 expressing Jurkat T cell coculture. The tumors in the irradiated and non-irradiated sites in the abscopal effective (Abs_eff_) group of both mouse models were regressed, proving the abscopal effect. The immunogenic effect in the Abs_eff_ group was associated with an expansion of T cell and other immune cells infiltration without changes in the CD39^+^ population in either the right or left tumors compared to control group. Furthermore, the abscopal ineffective (Abs_ineff_) group demonstrated lesser increase of T cells, decreased immune cell infiltration, and increased CD39-expressing Treg cells without suppression of tumor growth. In the coculture with PD-1-expressing Jurkat T cell, Ce6-PDT efficiently suppressed the PD-1/PD-L1 interactions by increasing the proliferation and cytotoxic activity of CD8^+^ T cells while decreasing CD39-expressing Treg cells in a dose-dependent manner. Likewise, the inhibition of PD-1/PD-L1 interactions was also correlated with the increased production of IL-2 and Granzyme B. Our findings imply that Ce6-PDT is a promising immunotherapy with the potential to improve the abscopal effect.

## Introduction

Photodynamic therapy (PDT) has become a prominent technique for cancer treatment due to its inherent advantages, such as a high degree of selectivity, less invasiveness, and minimal systemic toxicity^1,2^. This therapy employs laser irradiation to transform molecular oxygen into tumor-killing reactive oxygen species (ROS). As a result, ROS can directly kill tumor cells by triggering necrosis or apoptosis, and it can impair the tumor vasculature by depriving it of oxygen and nutrients^3,4^. Further, PDT may also affect the immune system’s immunosuppressive or immunostimulatory functions^5^. For many years, the explanation for how PDT affected cancer was only through local effects^6^. However, a new research has demonstrated that PDT also has the potential to eliminate metastatic and disseminated tumor cells by promoting antitumor immunity. PDT induces an effective anticancer immune response by forcing tumor cells to die in an immunogenic manner^7,8^. These dying tumor cells often release or expose damage-associated molecule patterns (DAMPs) as ‘immunogenic signals’, that attract antigen-presenting cells (APCs) and are subsequently presented to CD8^+^ T cells, inducing a long-lasting antitumor immune response^8^. Through PDT, antitumor responses to both primary and distant cancers are connected by immunogenic cell death (ICD), dramatically increasing the immunogenicity of dying cancer cells, and causing controlled immune system activation^9^. Such immune responses generate the abscopal effect, a phenomenon where local irradiation induces distant contralateral non-treated tumors to regress^10^.

The abscopal effect has been observed in patients with metastatic cancer treated with PDT alone or in conjunction with other therapy; however, it is rare. In the phase II clinical trial, patients with intraperitoneal tumors that primarily originated from ovarian cancer responded favorably to PDT with Photofrin (2.5 mg/kg) treatment^11^. According to Mortano et al., local recurrence and development of lung metastasis in clinical cases of Feline Injection-Site Sarcoma were significantly decreased after administration of combined PDS (Photodynamic Surgery) and PDT with acridine orange, reflecting the occurrence of an abscopal effect^12^. Furthermore, Kabingu et al., reported that enhanced cytolytic activity of splenocytes and infiltration of CD8^+^ lymphocytes were responsible for the remission of tumors at the distant site induced by local treatment of Photofrin-mediated PDT^13,14^.

Some preclinical trials have demonstrated benefit in other malignancies; the abscopal effect appears to be more common in tumors that are more immunogenic, such as melanoma, breast, lung, and liver cancers^15^. Despite the fact that PDT has been licensed and used for many different cancer types, the melanin pigmentation of melanoma limits the therapeutic efficiency of many existing photosensitizers like Photofrin, as melanin absorbs light at wavelengths between 300 and 600 nm^16^. However, PDT with photosensitizers, like Chlorin e6 (Ce6), is an effective and well-tolerated method of melanoma treatment because its absorption peak lies in the long-wave spectral region of 660 nm, which increases its ability to penetrate in biological tissues^17,18^. Since T cell dysfunction serves as a means of cancer immune escape, targeting the expression of PD-1 and PD-L1 immune checkpoints is an essential tactic for enhancing immunity against cancers^19^. Therefore, tumor survival and development may be affected by the PD-1/PD-L1 signaling axis^20^. The transmembrane protein PD-1 is known to be expressed on the surface of a variety of immune cells, including activated T cells like CD4^+^ and CD8^+^ T cells (cytotoxic T lymphocytes, or CTLs), while PD-L1 is expressed in tumor cells, and its interaction with PD-1 prevents the immune system-mediated killing of cancer cells^21^. As a result, T cell activation in peripheral tissues and the tumor milieu is suppressed by the molecular receptor PD-1, which further contributes to immune evasion^22,23^. Immune checkpoint inhibitors (ICIs) authorized by the FDA such as pembrolizumab, nivolumab, and cemiplimab target PD-1 or PD-L1 to a number of human tumors and have recently been shown to be effective in immunotherapy^24,25^. As opposed to monoclonal antibodies, small molecule inhibitors like BMS-200 and BMS-202 also offer a number of advantages in addressing these issues^26,27^.

Therefore, we investigated the immunogenic variables that affect the abscopal effect of Ce6-PDT. A bilateral subcutaneous melanoma and pancreatic tumor mouse model was employed in this study to test Ce6-PDT for the treatment of both local and distant tumors. Higher CD8^+^ T cell and macrophage peritumoral infiltration are linked to the antitumor effects of Ce6-PDT. PD-1/PD-L1 checkpoint inhibition has not yet been explored for Ce6-PDT. Hence, these findings suggest Ce6-PDT as a new immunotherapeutic strategy that targets primary tumors and induces systemic antitumor immunity by targeting the PD-1/PD-L1 interactions.

## Result

### Ce6-PDT elicited the inhibition of tumor growth

**Figure 1:**
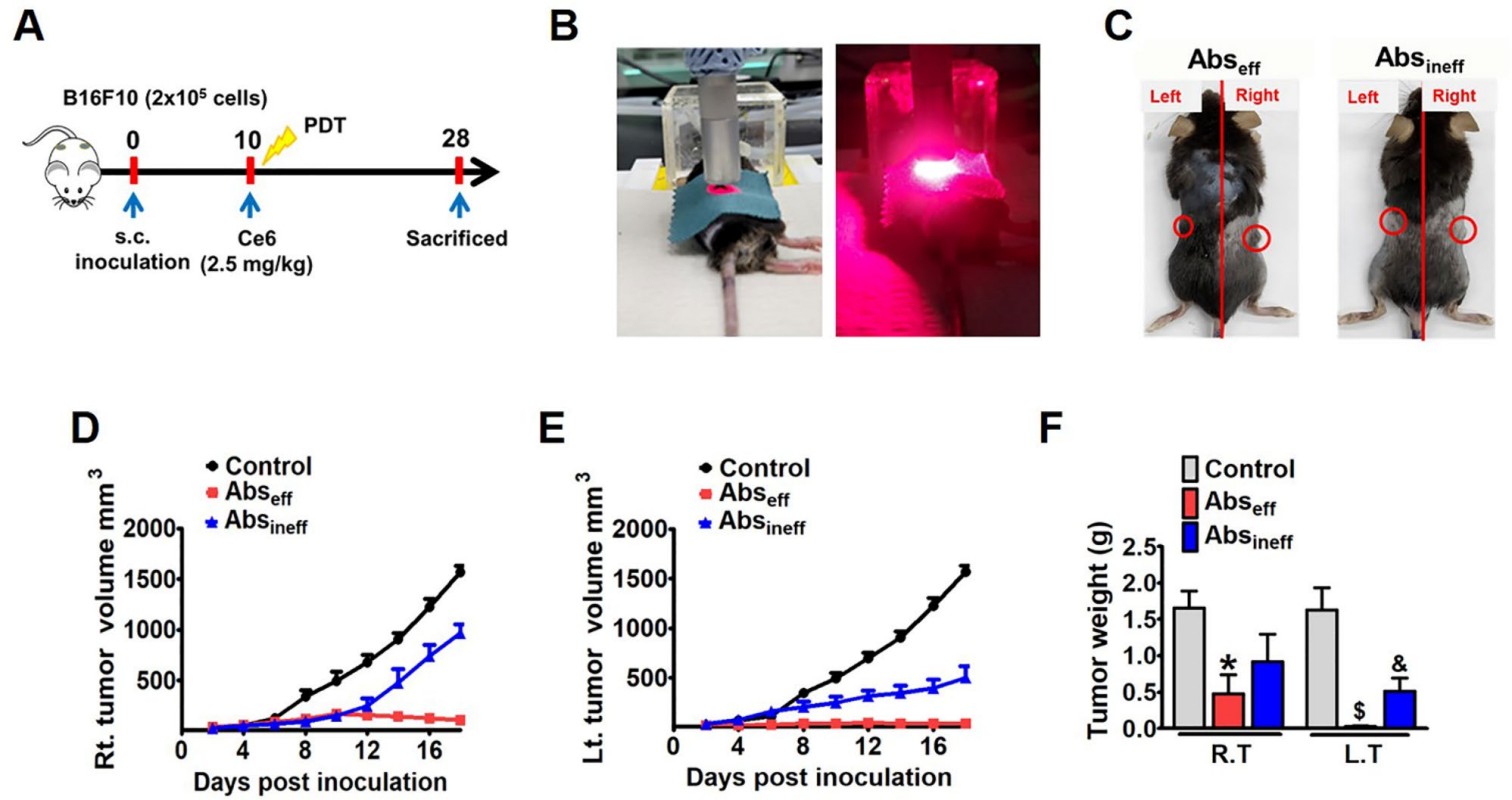
Abscopal antitumor effects in the melanoma mouse model. (**A**) Experimental scheme, (**B**) C57BL/6 mice were inoculated with B16F10 cells, received an intravenous injection of Ce6 (2.5 mg/kg) followed by irradiation using a diode laser (λ = 660 nm) at a rate of 100 J/cm^2^ for 8 min 20 s. (**C**) Mice were divided into Abs_eff_ and Abs_ineff_ groups on the basis of the abscopal effect with Ce6-PDT. (**D**) Right tumor (RT) volume, (**E**) left tumor (LT) volume, (**F**) right and left tumor weight in the control, Abs_eff_ and Abs_ineff_ groups respectively. Data are from an experiment representative with n = 3 in the control, n = 3 in the effective, and n = 4 in the ineffective group. *P < 0.05 compared to right control tumor.  ^#^*P* < 0.05 compared to right tumor of the abscopal effective group, ^$^*P* < 0.05 compared to left control tumor, and ^&^*P* < 0.05 compared to left tumor of the abscopal effective group (by one-way ANOVA with Tukey's post hoc test for multiple comparisons).

**Figure 2:**
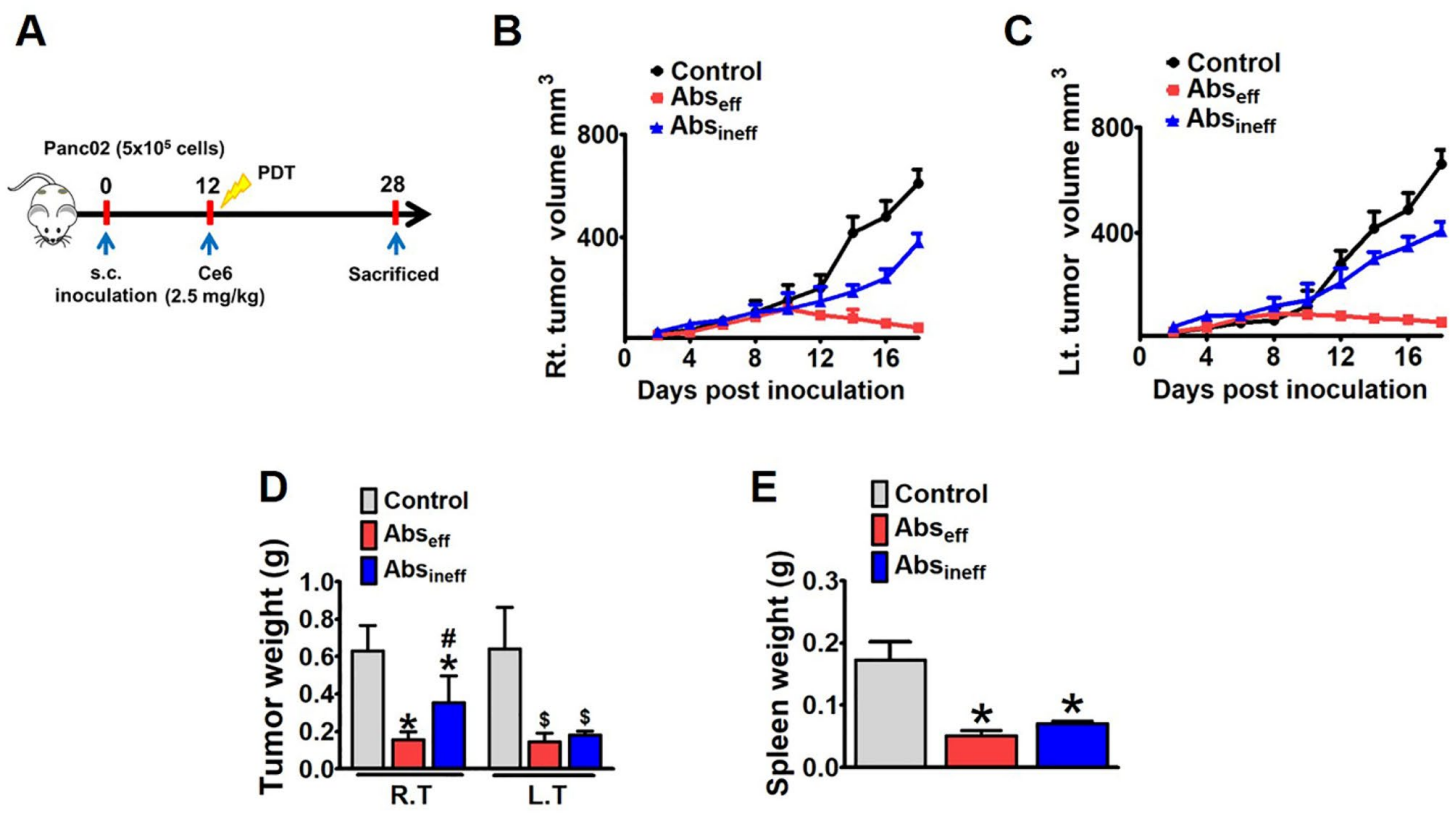
Abscopal antitumor effects in the pancreatic tumor mouse model. Panc02 cells of 5 × 10^5^ cell density were injected in the right and left flanks of mice. On the 12^th^ day, Ce6 (2.5 mg/kg) was injected intravenously and after 3 h followed by irradiation using a diode laser (λ = 660 nm) at a rate of 100 J/cm^2^ for 8 min 20 s. (**A**) The experimental scheme, (**B**) right tumor volume (RT), (**C**) left tumor (LT) volume, (**D**) tumor weight and (**E**) spleen weight in the control, Abs_eff,_ and Abs_ineff_ groups. Data are from an experiment representative with n = 3 in the control, n = 4 in the effective, and n = 4 in the ineffective group. *P < 0.05 compared to right control tumor. **#P** < 0.05 compared to right tumor of the abscopal effective group, ^$^*P* < 0.05 compared to left control tumor, and ^&^*P* < 0.05 compared to left tumor of the abscopal effective group (by one-way ANOVA with Tukey's post hoc test for multiple comparisons).

To investigate the Ce6-PDT-mediated antitumor abscopal effect, two bilateral tumor mouse models were developed using B16F10 melanoma and the Panc02 pancreatic cancer cells. After injecting Ce6 (2.5 mg/kg) followed by local irradiation to right tumor, we tested the ability of Ce6-PDT to prevent tumor growth in both irradiated right and non-irradiated left tumors from day 11 up to day 28 (Figs. 1A,B, and 2A). All PDT-treated animals initially had tumor shrinkage in the irradiated region; however, some mice later developed tumor regrowth due to the aggressiveness of B16F10 tumors. The Abs_eff_ and Abs_ineff_ groups were separated based on the tumor-specific efficacy of PDT and abscopal effect (Fig. 1C). Our findings indicated that Ce6-PDT exerted a dramatic suppression on the irradiated tumor volume of the Abs_eff_ group as compared to the Abs_ineff_ group, in Fig. 1D,E. Compared to tumors in the control and Abs_ineff_ group, the non-irradiated melanoma tumors of the Abs_eff_ group also displayed much lower tumor weight (Fig. 1F). The Abs_ineff_ group showed larger tumor weight on both the left and right sides as compared to the Abs_eff_ group. A 2.5 mg/kg dosage of Ce6-PDT also reduced tumor volume and weights in both irradiated and non-irradiated pancreatic tumors in the Abs_eff_ group (Fig. 2B,C,D) as compared to that in Abs_ineff_ and control mice. The spleen weights of the pancreatic tumor-bearing mice of the Abs_eff_ and Abs_ineff_ groups were also assayed. Similar to the tumor weight changes, the spleen of the Abs_eff_ group showed lesser weight than those of the Abs_ineff_ group and control group (Fig. 2E). The current investigation suggested that in both the effective and ineffective groups, the tumor volume, weight of the tumor and spleen showed similar patterns. Thus, Ce6-PDT suppressed growth of both irradiated right and non-irradiated left tumors in the Abs_eff_ group.

### Ce6-PDT-induced accumulation of tumor-infiltrating T cells in the Abs_eff_ tumors of the B16F10 mouse model

**Figure 3:**
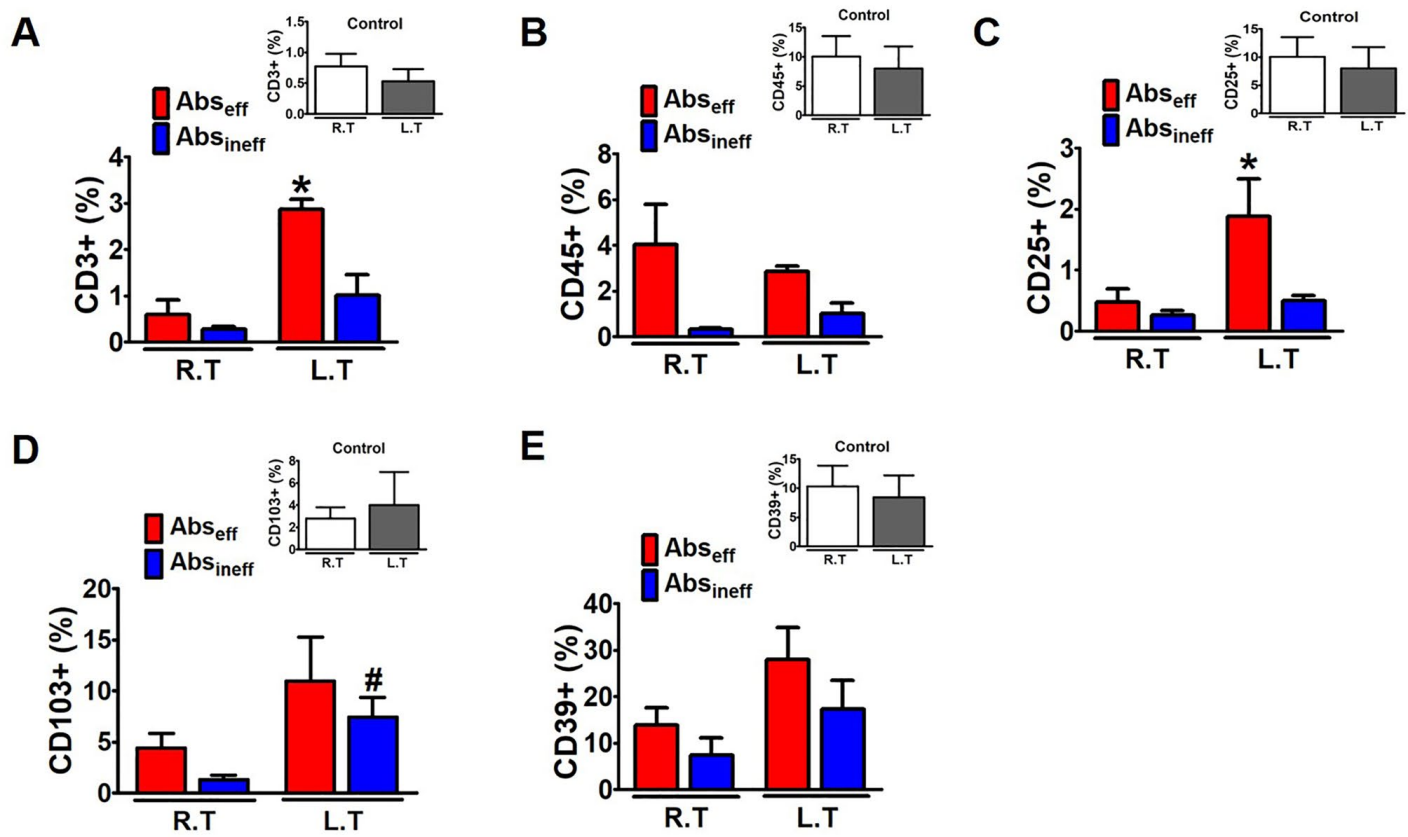
Enhanced accumulation and activation of T cells by Ce6-PDT in the melanoma mouse tumors. (**A**-**E**) Flow cytometry analysis to count and estimate the intratumoral fraction of (**A**) CD3^+^, (**B**) CD45^+^, (**C**) CD25^+^, (**D**) CD103^+^, and (**E**) CD39^+^ T cells, isolated from the irradiated right and non-irradiated left tumors in control, Abs_eff_ and Abs_ineff_ group. After 28 days of tumor cell injection, T cells in tumor tissues were isolated from B16F10 tumor-bearing mice. Data are from an experiment representative with n = 3 in the control, n = 3 in the abscopal effective, and n = 4 in the abscopal ineffective group. *p < 0.05 compared to irradiated right tumors in abscopal effective group. #*p* < 0.05 compared to irradiated right tumors in abscopal ineffective group (by one-way ANOVA with Tukey's post hoc test for multiple comparisons).

Efficient induction of T helper cells and cytotoxic T cells is essential for persistent suppression of tumors to attain PDT and its abscopal effect^28^. We measured the flow-cytometric analyses of T cells and their subtypes in control, Abs_eff_, and Abs_ineff_ tumors of the irradiated right and non-irradiated left regions. We assayed the percentages of tumor T-cell infiltration of CD3^+^ , CD45^+^ , CD25^+^ , CD103^+^ , and CD39^+^ (Figs. 3A–E), to evaluate the systemic immune response after the primary tumors were subjected to PDT. After concurrent with reduced tumor growth, the Ce6-PDT treatment increased the percentage of CD3^+^ CD4^+^ tumor-infiltrating T cells and CD45^+^ , a leukocyte common antigen, indicating an increased immune activation (Fig. 3A,B). Ce6-PDT also resulted in the decrease of the irradiated tumor volumes, suggesting stronger T cell responses. Allografts in the Abs_eff_ group exhibited considerably lower frequencies of regulatory CD25^+^ T cells in both the irradiated and non-irradiated tumors compared to the control, which signifies abrogation of their immune inhibitory activity (Fig. 3C). In the Abs_eff_ group, the irradiated right tumor showed a low frequency of CD8^+^ CD103^+^ and CD8^+^ CD39^+^ T cells, but higher levels in the non-irradiated tumor (Fig. 3D,E). However, in the Abs_ineff_ group, a decreased infiltration of CD3^+^ , CD45^+^ T cells was noted, thus indicating decrease in T cell function and poor prognosis of the tumor. We have also provided the gating scheme to determine the T cells and their subtypes in the supplementary file (SI-S3). These findings show that Ce6-PDT elicits host immunological responses through an increase in number of T cells. However, the abscopal ineffective tumors may have a hindrance in the tumor microenvironment that does not allow immune cells to migrate into distant tumor areas and results in lower T cell activation ^29,30^.

### Ce6-PDT promoted the immune cell distribution in the Abs_eff_ tumors of the melanoma cancer mouse model

**Figure 4:**
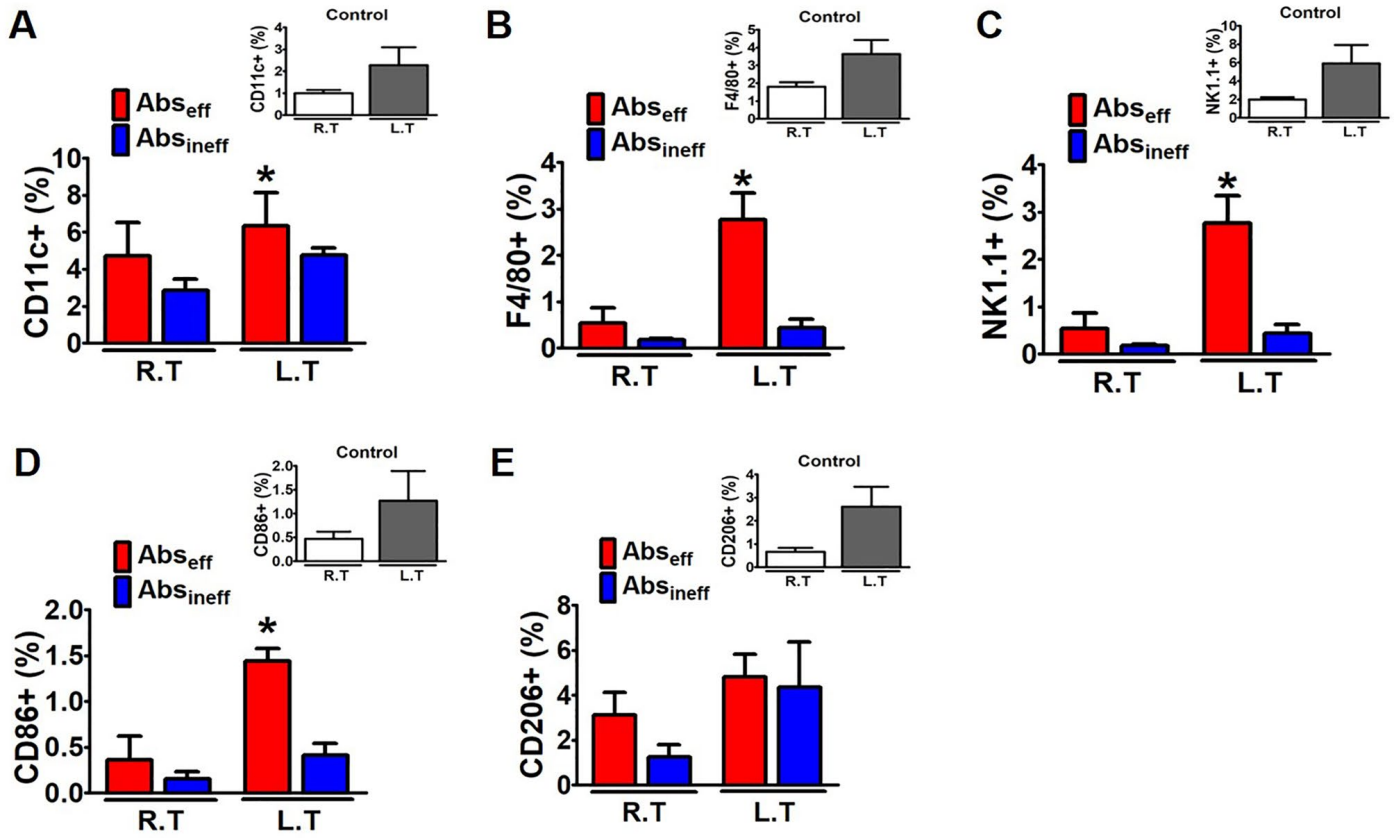
Flow cytometric analysis of the immune cell in the irradiated and non-irradiated tumor of melanoma mouse tumors**.** Percentages of (**A**) CD11c^+^, (**B**) F4/80^+^, (**C**) NK1.1^+^ (**D**) CD86^+^, and (**E**) CD 206^+^ in irradiated and non-irradiated tumor in control, Abs_eff_ group, and Abs_eff_ group. Data are from an experiment representative with n = 3 in the control, n = 3 in the effective, and n = 4 in the ineffective group. *p < 0.05 compared to irradiated right tumors in abscopal effective group. #*p* < 0.05 compared to irradiated right tumors in abscopal ineffective group (by one-way ANOVA with Tukey's post hoc test for multiple comparisons).

Macrophages and other immune cells were similarly explored using flow cytometry in the right and left tumors of the melanoma mouse model. We then evaluated the population and status of macrophages in the Abs_eff_ tumors to provide additional insight into the activation mechanism of the immune response produced by Ce6-PDT in vivo. We analyzed the percentages of CD11c, F4/80 (macrophage-monocyte marker), NK 1.1, CD86, and CD206, which are necessary for the induction of adaptive immunity, in the Abs_eff_ and Abs_ineff_ groups (Fig. 4A–E). We noted that CD11c^+^ and F4/80^+^ macrophage populations were increased in the irradiated right tumors, and the increased patterns were persisted in the non-irradiated left tumors in the Abs_eff_ group (Fig. 4A,B). Similarly, in the Abs_eff_ group, NK cells were increased in the non-irradiated tumors as compared to the ineffective group (Fig. 4C). However, the percentages of CD11c^+^ , F4/80^+^ macrophage, and NK cells in the Abs_ineff_ group were decreased. A key component of tumor immunity is the expression of CD86^+^ on macrophages and dendritic cells, which are essential for antigen presentation to T cells. The percentage of CD86^+^ and CD206^+^ macrophages was significantly higher in the non-irradiated tumors compared to the irradiated tumor of the Abs_eff_ group (Fig. 4D and E). The total number of immune cells in the abscopal effective tumor was significantly higher, which is consistent with the functioning of T cells and the pronounced abscopal responses in the Abs_eff_ group as compared to the Abs_ineff_ group.

### Ce6-PDT increased the intra-tumoral density of T cells in the pancreatic cancer mouse model

**Figure 5:**
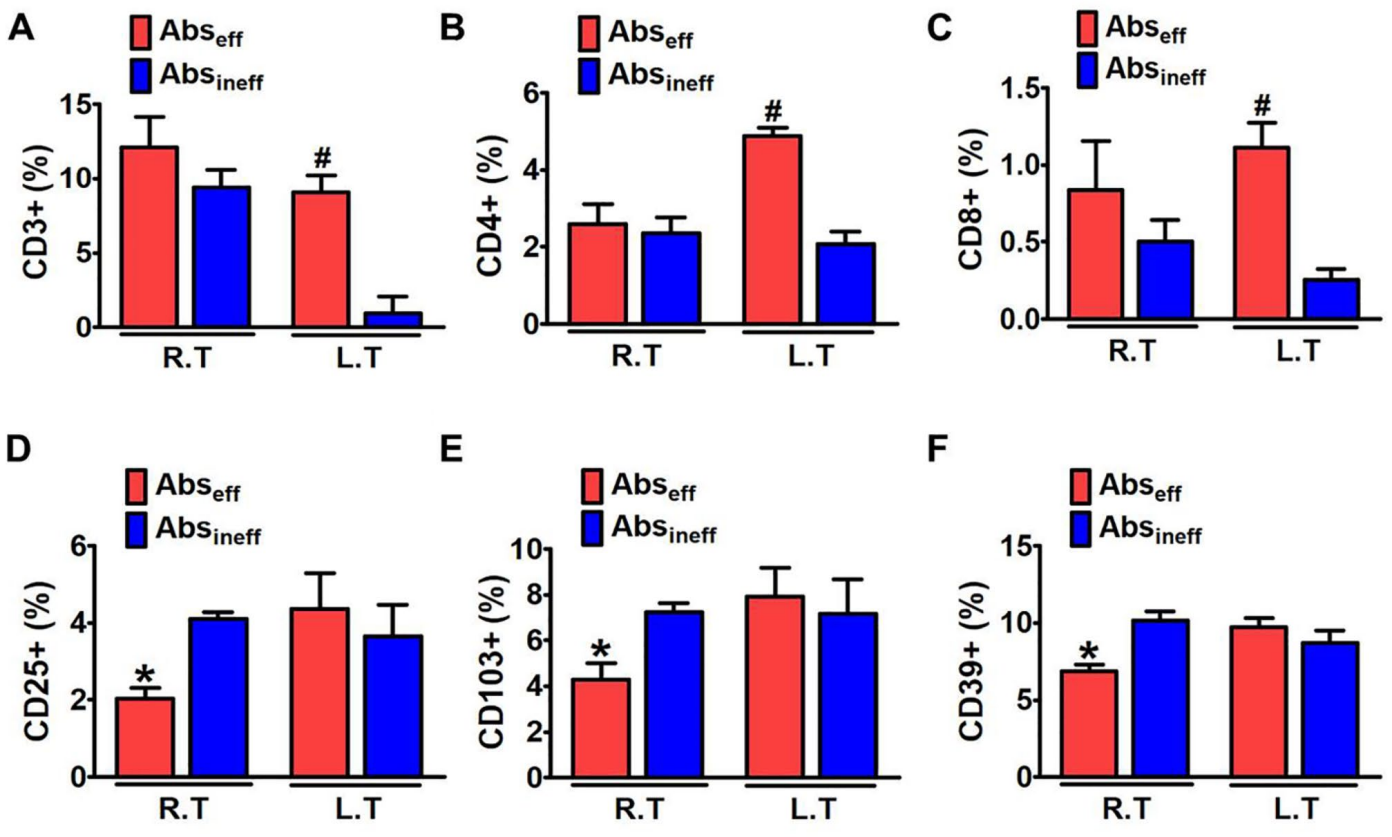
Enhanced accumulation and activation of T cells by Ce6-PDT in the pancreatic mouse tumors. (**A**-**E**) Flow cytometry analysis to count and estimate the intratumoral fraction of (**A**) CD3^+^, (**B**) CD4^+^, (**C**) CD8^+^, (**D**) CD25^+^ (**E**) CD103^+^, and (**F**) CD39^+^ T cells, isolated from the irradiated right and non-irradiated left tumor in Abs_eff_ group and Abs_ineff_ group. After 28 days of tumor cell injection, T cells within tumors were isolated from Panc02 pancreatic cancer bearing mice. Data are from an experiment representative of three mice in the effective and four mice in the ineffective group. *P < 0.05 compared to right tumor. **#P** < 0.05 compared to left tumor (by one-way ANOVA with Tukey's post hoc test for multiple comparisons).

We also focused on the following group of markers to investigate the infiltration of various T cell subpopulations such as CD3^+^ , CD4^+^ , CD8^+^ , CD25^+^ , CD103^+^ , and CD39^+^ in the pancreatic cancer model with Ce6-PDT treatment (Fig. 5A–F). The pancreatic tumors in the irradiated right and non-irradiated left sides of the Abs_eff_ group showed higher CD3^+^ T cells compared to the Abs_ineff_ group. CD4^+^ helper and CD8^+^ cytotoxic T cells make up the majority of T cell populations in the tumors (Fig. 5B and C). A significantly higher CD3^+^ T cell infiltration is found in the left tumors compared to the right tumors (Fig. 5A). However, lesser effects were detected in the Abs_ineff_ group. We further analyzed CD25^+^ , CD103^+^ , and CD39^+^ levels in both Abs_eff_ and Abs_ineff_ groups (Fig. 5D–F). The pancreatic tumors on the right side revealed a lower percentage of CD25^+^ , CD103^+^ , and CD39^+^ compared to the non-irradiated left side in the Abs_eff_ group while the increased percentages of CD25^+^ , CD103^+^ , and CD39^+^ were observed in the Abs_ineff_ group. Thus, similar to the melanoma mouse model, Ce6-PDT elicits a positive abscopal effect through the inhibition of Treg cells that resulted in the activation of CD4^+^ and CD8^+^ T cells. However, the population of T cells in the spleen of the Abs_eff_ and Abs_ineff_ group did not show any difference (SI-1, Fig. S1).

### Ce6-PDT promoted the intratumoral immune cell distribution in the pancreatic cancer mouse model

**Figure 6:**
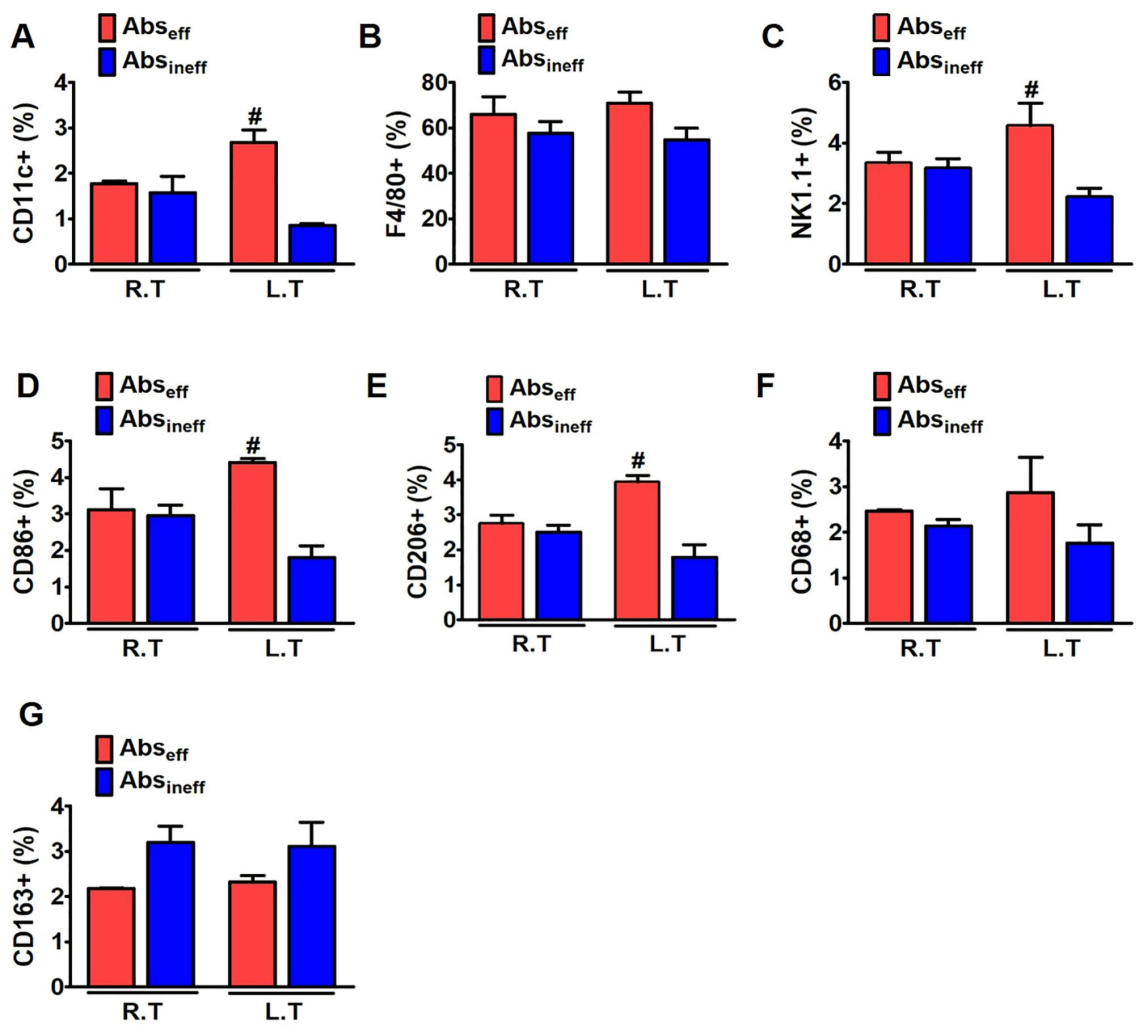
Flow cytometric analysis of the immune cells in the irradiated and non-irradiated tumor of pancreatic mouse tumors. Percentages of (**A**) CD11c^+^, (**B**) F4/80^+^, (**C**) NK1.1^+^ (**D**) CD86^+^ (**E**) CD 206^+^ (**F**) CD68^+^, and (**E**) CD163^+^ in (A) 24 and (B) 48 h from the irradiated and non-irradiated pancreatic tumor in the Abs_eff_ group and Abs_ineff_ group. Data are from an experiment representative of three mice in the effective and four mice in the ineffective group. *P < 0.05 compared to right tumor **#P** < 0.05 compared to left tumor (by one-way ANOVA with Tukey's post hoc test for multiple comparisons).

Focusing on the role of innate immune system with Ce6-PDT treatment in C57BL/6 mice, we examined the percentages of macrophages, dendritic cell, and NK cell populations in the tumor tissues by FACS analysis (Fig. 6A–G). CD11c^+^ (macrophage cells) were increased in the irradiated tumors and non-irradiated tumors in the Abs_eff_ group compared to the Abs_ineff_ group (Fig. 6A). The results showed that the percentage of F4/80 and NK1.1 cells increased in both the irradiated and non-irradiated tumors of the Abs_eff_ group (Fig. 6B,C). Accordingly, an increased CD86^+^ population revealed increased maturation of dendritic cells, following the same pattern as Abs_eff_ group of the melanoma model (Fig. 6D). In the Abs_eff_ group, CD86^+^ was found to be higher in the non-irradiated tumors than in the irradiated tumors, whereas the Abs_ineff_ group showed a different result. CD206^+^ and CD68^+^ were also increased, indicating that PDT treatment triggers macrophage infiltration and inflammatory response in the distant tumor sites (Fig. 6E,F). The Ce6-PDT treatment significantly increased tumor-associated CD163 + M2 macrophage in the ineffective group, pointing out the poor prognosis of the tumor (Fig. 6G). Therefore, these data indicated that the percentages of myeloid cells were increased in the effective group but decreased in the ineffective group. In the spleens of effective and ineffective groups, we found similar patterns of immune cell increase for CD11c^+^, F4/80^+^, NK^+^, CD86^+^, and CD206^+^ as shown in the tumors (SI-2, Fig. S2).

### Ce6-PDT enhanced T cell-associated cytokines by inhibiting PD-1/PD-L1 checkpoint

**Figure 7:**
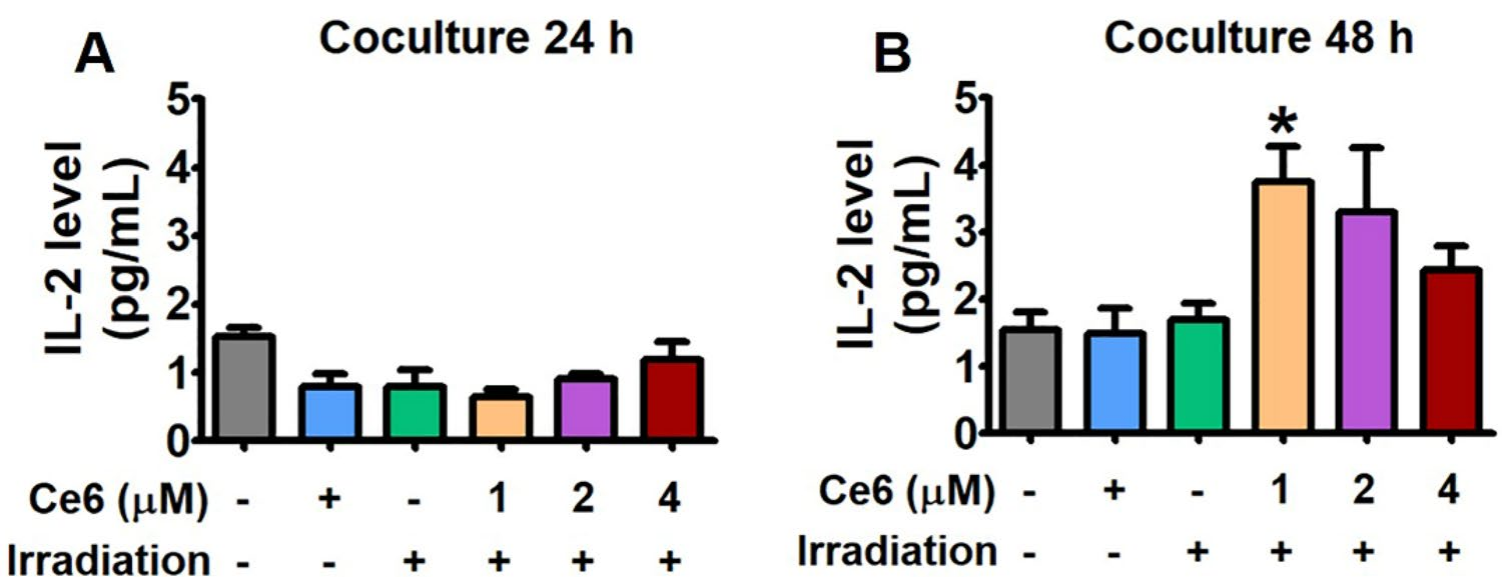
Effect of Ce6-PDT on IL-2 cytokine release in Jurkat T cells and MC38 colon cancer cells coculture model. Cell culture media was analyzed to measure IL-2 level by cytokine ELISA in (**A**) 24 and (**B**) 48 h. The concentration of Ce6 only is 4 μΜ. Data are presented as mean ± S.E. of three representative independent experiments. *P < 0.05 compared to vehicle-treated control. (by one-way ANOVA with Tukey's post hoc test for multiple comparisons).

To better understand the effect of Ce6-PDT on T-cell receptor (TCR) activation, we used coculture systems with human PD-1-expressing Jurkat T cells that expressed nuclear factor of activated T cells (NFAT)-derived luciferase reporter (hPD-1/NFAT Jurkat T cells) and human PD-L1-expressing APC/CHO-K1 cells engineered to activate cognate TCR (hPD-L1/TCR CHO-K1 cells). The cell viability of hPD-1 (Jurkat T cells) and hPD-L1/TCR CHO-K1 for 24 h following Ce6-PDT is provided in the SI-4, Figs. S4A,B. We predicted that Ce6-PDT would have an effect on the binding activities of PD-1 and PD-L1. Therefore, a competitive ELISA was used to examine Ce6-PDT’s effect in PD-1 and PD-L1 interaction. IL-2, as a T cell growth and proliferation factor, can induce the expansion of tumor-infiltrating cytotoxic T cells and NK cells. Because IL-2 plays an important role in the enhancement of T cell-mediated antitumor immune responses^31^, we investigated whether Ce6-PDT induces IL-2 release for tumor regression. In order to detect the ability of Ce6-PDT to improve T cell activity, the secretion of IL-2 was analyzed in the coculture system (Fig. 7A,B). In the system with escalating dosages (1–4 μM) of Ce6 followed by PDT for 48 h in hPD-1 effector and hPD-L1/TCR CHO-K1 cells, released IL-2 was increased, suggesting T cells activation by Ce6-PDT. Ce6-PDT at 1 μΜ elevated the levels of IL-2 up to about twofold higher than the untreated group, which then decreased from 2 to 4 μΜ in 48 h. However, for 24 h after Ce6-PDT treatment, IL-2 secretion was not detectable. These results suggest that Ce6-PDT effectively enhanced T cell immune function by inhibiting the PD-1/PD-L1 axis in 48 h.

### Tumor-specific CD8^+^ T cells upregulated granzyme B

**Figure 8:**
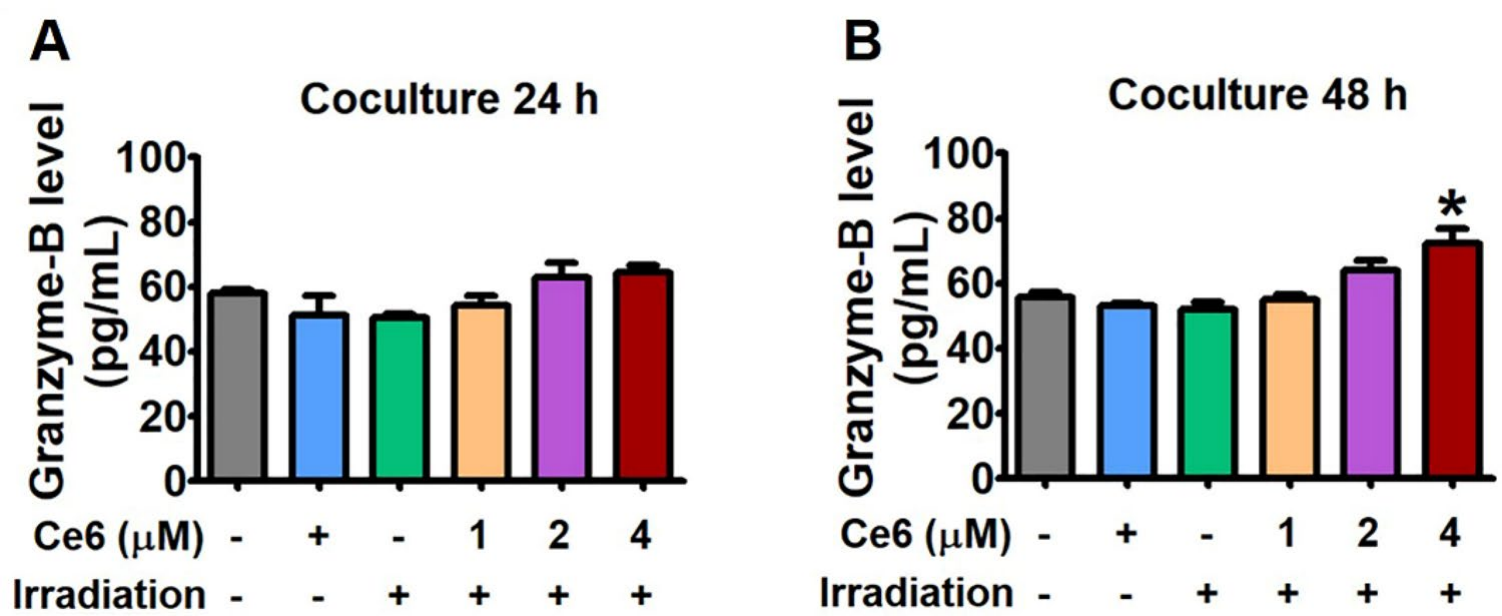
Inhibition of PD-1/PD-L1 checkpoint by Ce6-PDT and increased granzyme B production. Cell culture media was analyzed to measure granzyme level by cytokine ELISA in (**A**) 24 and (**B**) 48 h. The concentration of Ce6 only is 4 μΜ. Data are presented as mean ± S.E. of three representative independent experiments. *P < 0.05 compared to vehicle-treated control. (by one-way ANOVA with Tukey's post hoc test for multiple comparisons).

In determining the level of granzyme B, we also evaluated the degranulation of CD8^+^ T cells from the cocultures. The CD8^+^ T cells that were treated with 2 and 4 μM of Ce6 and then exposed to 660 nm light exhibited elevated levels of granzyme B in 24 and 48 h respectively, as compared to the untreated and the Ce6 only (4 μM)-treated group (Fig. 8A,B). Ce6-based PDT at doses of 1 and 2 μM did not differ in any way from the untreated control. The levels of granzyme B in the tumor-specific CD8^+^ T lymphocytes were lower during 24 h incubation but were found to be increased after 48 h of incubation. These findings suggest that Ce6-PDT can cause CD8^+^ T cells to release granzyme B, which further trigger the death of targeted tumor cells and increase immune responses.

### Ce6-PDT blockade of PD-1/PD-L1 interaction enhanced CD8^+^ T cell activities and reduced CD39^+^ T cells

**Figure 9:**
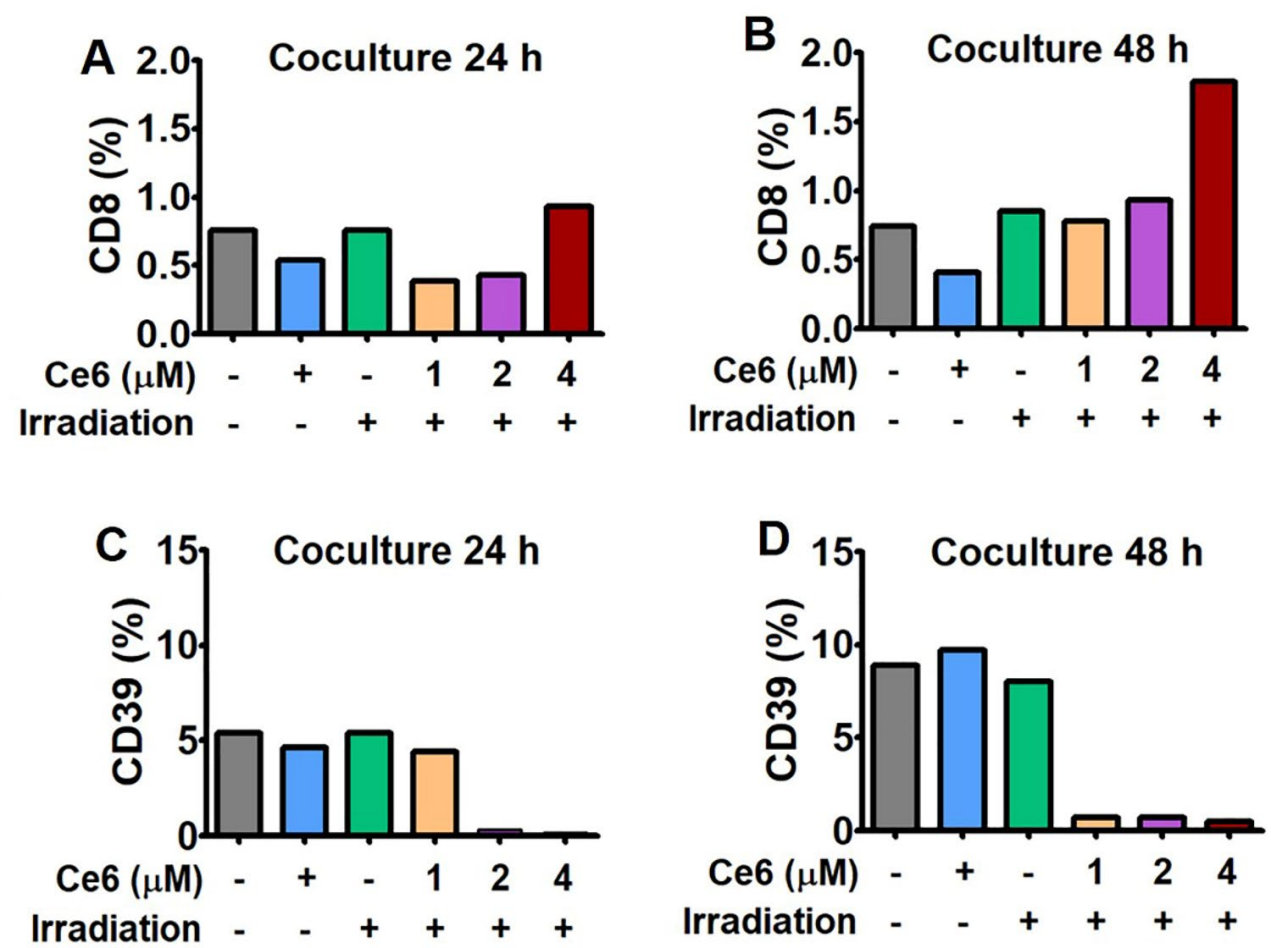
Augmentation of cytotoxic T cells and deduction of CD39^+^ Treg cells through the blockage of PD-1/PD-L1 interaction by Ce6-PDT. PD-L1 aAPC/CHO-K1 cells were plated and incubated at 37 °C for 20 h prior to the addition of PD-1 effector cells. Cells were treated with an increasing concentrations of Ce6 (1–4 µM) followed by PDT. The concentration of Ce6 only is 4 μΜ. PD-1/PD-L1 immune check-point blockage by Ce6-PDT was analyzed to measure the CD8^+^ T cells and CD39^+^ T cells (**A**), (**C**) in 24 h and (**B**), (**D**) in 48 h of coculture respectively. Data are presented as the means only.

The ability of Ce6-PDT to enhance CD8^+^ T cell activation was assessed using a PD-1/PD-L1 blockade assay. In this study, Ce6 was utilized at concentrations ranging from 1 to 4 μΜ, and of these, 4 μΜ demonstrated the highest PD-1/PD-L1 blockage with enhanced CD8^+^ T cell functional activity. In both the 24 and 48 h of coculture, CD8^+^ T cell functional activity at 4 μΜ was significantly higher than at other Ce6 doses (Fig. 9A,B). When compared to the control, Ce6-PDT (4 μM) treatment for 48 h enhanced T cell functional activity by 2.2 fold. We also examined the expression of CD39^+^ T cells in Ce6-PDT-treated cells (Fig. 9C,D). PDT with Ce6 of 2 and 4 μΜ significantly decreased the level of CD39^+^ at 24 h coculture compared to untreated, Ce6 only, and light only-treated cells. However, at 48 h coculture, the level was significantly declined with Ce6 doses from 1 to 4 μΜ. The PD-1/PD-L1 blockage achieved by Ce6-PDT produced antitumor effects by increasing the cytotoxic function of CD8^+^ T cell while decreasing the infiltration of CD39^+^ T cells.

## Discussion

PDT has been considered useful for managing and eliminating local lesions in several malignant cancers^32^. The systemic impact of the abscopal effect by PDT is becoming more and more of a focus^3^. To test the Ce6-PDT-mediated abscopal effect, we first established two immunogenic melanoma and pancreatic cancer mouse models (B16F10 and Panc02 cells). We discovered that in these models, Ce6-PDT generated the abscopal effect. Multiple studies have demonstrated that PD-1/PD-L1 targeted therapy presents a possible immunotherapy strategy for cancer by suppressing various stages of the immunological response between T cells and tumors^9,33^. The current study found that Ce6-PDT induced T cell activation and antitumor immunity by blocking PD-1 and PD-L1. However, the inhibitory effects of Ce6-PDT on the immune checkpoint are currently unknown and hence need further investigation.

According to Ebner et al. (2017), the abscopal effect is a naturally occurring ability that can be activated and amplified by therapies such as immune treatment or radiation and depend on the actions of specific but undiscovered host-tumor epigenetic factors^34^. This supports our study's discovery that Ce6-PDT-treated mice had different abscopal outcomes, which led to the division of these animals into two main groups: an abscopal effective group (tumors on both irradiated and non-irradiated sides decreased), and an abscopal ineffective group (tumors on both sides did not decrease). The noteworthy outcomes in the Abs_eff_ group may be attributed to Ce6-PDT's ability to modulate the tumor surroundings, which caused irradiated tumor cells to produce tumor antigens and immunogenic cell death. These antigens might be picked up by antigen-presenting cells like dendritic cells, which is correlated with increased infiltration of CD11c^+^ , F4/30^+^ , and co-stimulatory molecules like CD86^+^ in the tumor of the Abs_eff_ group. It is well known that activated CD4^+^ T cells might assist CD8^+^ T cells and further support the CD8^+^ effector for its memory formation and maintenance. We found that these CD8^+^ effector memory T cells then produce molecules such as granzyme A, granzyme B, perforin, etc., which have cytotoxic effector functions and assist in slowing the growth of the distal tumor in the effective group^35,36^. Our study suggests that the innate immune system was involved, which most likely made it easier for neutrophils, dendritic cells, and macrophages to gather. Several studies have shown that PDT stimulates the immune system in many ways, including the release of tumor-associated antigens (TAAs) and immune-stimulatory molecules from tumors, which may activate and trigger an anti-cancer immune response^37,38^. Although PDT has been shown to activate both humoral and adaptive cell-mediated immunity, CD8^+^ T cells are primarily responsible for PDT's immunological effects^39^.

Our findings demonstrated that the infiltration of CD8^+^ T cells was enhanced by Ce6-PDT in the irradiated and non-irradiated tumors of the Abs_eff_ group. However, T cells were exhausted in the non-irradiated tumor of the Abs_ineff_ group and failed to elicit an anticancer immune response. In the non-irradiated left tumor, we observed a reduction in the percentage of CD3^+^, CD4^+^, and CD8^+^ T cells after PDT in the ineffective group. The depletion of CD8^+^ T cells in the ineffective group indicates antitumor immunity is insufficient, as proliferation and activation of CD8^+^ T cells are too low and lack persistence due to the aggressiveness of tumors. The inability of the host CD8^+^ T cells to localize to a tumor might be due to the absence of sufficient immunogenic antigens for T cell recognition. These tumors might not have a high mutation rate in order to generate immunogenic neoantigens capable of activating CD8^+^ T cells^40^. According to previous study, tumors may have barriers in their milieu that prevent responding immune cells from migrating and penetrating into non-irradiated tumor^39^. Among the ineffective group, the composition of immune cells in the irradiated right tumor was predominantly changed by PDT, as characterized by fewer dendritic cells (DCs). Tumor-infiltrating immune cells represent the actual conditions of the tumor immune microenvironment. The immune cells such as macrophages (CD11c^+^ and F4/80^+^) were reduced in the ineffective group in comparison to the effective group, indicating that Ce6-PDT was unable to promote the sufficient release of tumor antigens (B16F10 and Panc02), which could not activate immune cells and further kill cancer cells. Therefore, in the ineffective group, decreased immune cells were associated with fewer T cells.

Previous research has shown that ligands such as PD-L1, are overexpressed in melanoma and pancreatic cancers^41,42^. Using the humanized PD-1 mouse model with hPD-L1 knock-in and the MC38 tumor-bearing PD-1 mouse model, we evaluated the antitumor effects of Ce6-PDT. According to our findings, Ce6-PDT-induced suppression of the PD-1/PD-L1 axis could stimulate increased activation of CD8^+^ T cells and improve antitumor activity. It is well known that IL-2 is also a central mediator of Treg suppression during immune response^43^. Therefore, a 2 to threefold reduction in the number of Tregs during PD-1/PD-L1 blockade could increase IL-2 release and the pool size of effector cells. Many studies have shown that combined IL-2 therapy and PD-L1 blockade can produce synergistic effects in treating human chronic inflammation and cancers^44^. Granzyme B is also a marker for T cell activation, which is known to be the product of either CD4^+^ or CD8^+^ T cells^45^. This cytokine's increased secretion in the coculture corresponds to increased activation of T cell after Ce6-PDT treatment with inhibition of PD-L1 and PD-1 check-point. In the study, Ce6-PDT increased the CD8^+^ T cell activity through IL-2 production at 2 μM concentration of Ce6. We also tested Ce6-PDT's capacity to increase T cell functional activity by preventing PD-1 and PD-L1 interactions. The results showed that Ce6-PDT increased CD8^+^ T cells and TCR signaling in a concentration-dependent manner with higher CD8^+^ T cell activity at 4 μM of Ce6. However, Ce6-PDT-induced PD-1/PD-L1 checkpoint inhibition reduced CD39^+^ expressing Treg cell infiltration in 2 and 4 μΜ doses of Ce6 after 24 and 48 h of incubation. Thus, the Ce6-PDT-associated decrease in PD-1/PD-L1 interaction is linked with increased tumor cells death in distant tumors with the enhanced frequency of CD8^+^ T cells through the release of IL-2, suppression of CD39^+^ T cell activity, and increased Granzyme B levels.

In conclusion, our findings for the first time suggest that Ce6-PDT can induce potent local and systemic antitumor immune responses. Antitumor effects of Ce6-PDT were achieved by inhibiting the PD-1/PD-L1 interaction, which significantly accelerated the abscopal effects. To our knowledge, this is also the first time the impact of Ce6-PDT on the abscopal effect has been evaluated by targeting PD-1 and PD-L1 interactions. The findings of this study may aid in the development of effective inhibitors that block PD-1 or PD-L1 immune checkpoints and improve the outcomes of Ce6-PDT. We believe that more research on Ce6-PDT in combination with other immune check-point inhibitors like pembrolizumab or nivolumab may provide a strategy to improve the abscopal effects and get a better prognosis.

## Methods

### Cell culture

B16F10 mouse melanoma cells procured from the Korean Cell Line Bank (KCLB; Seoul, Korea) were cultured in Dulbecco's Modified Eagle Medium (DMEM) with 10% heat-inactivated fetal bovine serum (FBS) and 1% Antimycin A. Panc02/Luciferase stable cell line, a mouse pancreatic epithelial cell line derived from an adenocarcinoma model was obtained from GenTarget (San Diego, CA, USA) and was cultured in DMEM supplemented with 10% FBS, at 37 °C, in a humidified incubator, under a 5% CO_2_ atmosphere. Every two days, the media was changed and sub-cultured until they reached 80–90% confluency.

Recombinant Jurkat T cells constitutively expressing human PD-1 and NFAT reporter genes (#60,535, hPD-1/NFAT Jurkat T cells) and recombinant CHO-K1 cells expressing human PD-L1 and TCR activator were both procured from BPS Bioscience. The Jurkat T cells were cultured in RPMI-1640 medium (GE Healthcare Life Sciences, Chicago, IL, USA) supplemented with 10% FBS and antibiotics (100 U/mL penicillin and 100 mg/mL streptomycin). The hPD-L1/TCR CHO-K1 cells were maintained in Ham's F-12 medium supplemented with 10% FBS and antibiotics. To maintain stable cells carrying genetic constructs, these cells were grown in a complete medium with geneticin (1 mg/mL) and hygromycin B (200 mg/mL). MC38 cells expressing human PD-L1 (hPD-L1 MC38 cells) were generated from C57BL/6 murine colorectal adenocarcinomas and were obtained from Shanghai Model Organisms Center, Inc. (Shanghai, China). The cells were cultured in DMEM supplemented with 10% FBS at 37 °C in a humidified incubator.

### Murine tumor model

Male C57BL/6 mice (5–6 weeks old) were procured from Orient Bio (Seongnam, Korea). Mouse models of bilateral melanoma tumors or pancreatic cancer models were established by injecting B16F10 (2 × 10^5^) or Panc02 (8 × 10^5^) subcutaneously into the right flank of mice (irradiated tumor) and the left flank (non-irradiated tumor) respectively of C57BL/6 mice. Every three days, the tumor dimensions were measured with digital calipers, and the volume was determined using the formula: volume = (W^2^ x L)/2 (W: short diameter; L: long diameter).

When the mean tumor volume reached 90 mm^3^, the mice were randomly assigned to various treatment groups (n ≥ 5 per group). The mice in the control group were administered normal saline (NS). The Ce6 solution was prepared by dissolving it in normal saline. In the irradiation group, PDT was conducted by injecting 100 μL of Ce6 into the tail vein for the choice of dose of 2.5 mg/kg, with a drug-PDT interval of 3 h and LED 660-nm illumination at 100 J/cm^2^. The explanation for the choice of a dose of 2.5 mg/kg Ce6 and the pharmacokinetic study for determining the drug interval time before light irradiation in PDT are given in the supplementary file (SI-S5, S6, Fig. S6). The vehicle-treated control, however, did not receive any light exposure (SI-S7, Fig. S7). After 28 days, all the mice were sacrificed, and their tumors were collected for further studies. All the images were taken by the first and second authors. Animal procedures were conducted in accordance with appropriate regulatory standards under the protocol IACUC #ds002205112-EUTO3, which was reviewed by the Institutional Animal Care and Use Committee of the Dongsung Cancer Center. These studies were carried out in compliance with the ARRIVE guidelines.

### Murine splenocytes or tumor-infiltrating T cells (TILs) isolation

Tumors from control, Abs_eff_ and Abs_ineff_ group were selected for tumor extraction. The single-cell suspension of mouse splenocytes was prepared by filtering through a 100-mm and 40-mm cell strainer (SPL Life Sciences, Pocheon , Korea) while red blood cells (RBCs) were removed with ammonium-chloride-potassium lysing buffer (Lonza, Basel, Switzerland). Further, tumors were digested with collagenase IV (0.2 mg/mL, Sigma-Aldrich) and DNase I (0.02 mg/mL, Sigma-Aldrich) for 30 min. Tumor tissues were then squashed through 70 m cell strainers with a syringe. TILs were extracted from the central layer of a 40–80% Percoll (Sigma-Aldrich) gradient centrifugation (run at 800 g for 20 min at room temperature without deceleration). CytoFLEX flow cell counter (Beckman) was used to examine TILs such as CD4^+^/CD5^+^, CD3^+^, CD8^+^/CD3^+^, CD25^+^/CD4^+^, CD45^+^, CD103^+^/CD8^+^, CD39^+^/CD8^+^, CD11c^+^, F4/80^+^, NK1.1^+^, CD86^+^, CD68^+^, and CD206^+^.

### IL-2 measurement assay

A sandwich ELISA was used to measure the amount of IL-2 released by activated T cells in the cell coculture supernatants according to the manufacturer's instructions. Briefly, the plates (0.32 cm^2^, # 3590, Corning) were coated with an anti-mouse IL-2 monoclonal antibody with 0.1 M sodium carbonate (pH 9.5) and treated overnight at 4 °C. The plates were then rinsed in Phosphate buffered saline with Tween 20 (PBS-T) and were blocked for 1 h at room temperature with PBS containing 10% FBS. Each well was treated for 1 h at room temperature with biotinylated IL-2 antibody and streptavidin-horse radish peroxidase (HRP). The relative absorbance was measured at 450 nm using a SpectraMax i3 microplate reader (Molecular Devices, San Jose, CA, USA).

### Granzyme measurement assay

According to the manufacturer’s instructions, the amount of Granzyme B produced by activated T cells in cell coculture was determined using culture supernatants that were gathered, spun free of cells, and frozen at -20 °C. The wells were blocked for 1 h at room temperature using PBS, 0.05% Tween-20, and 0.1% bovine serum albumin (BSA) after two PBS washes. After adding the samples for 2 h at room temperature, wells were washed and further 1 g/mL Granzyme B-biotin was added for 1 h. Wells were washed and were incubated in 1 g/mL of streptavidin-HRP for 1 h. Wells were washed following the addition of streptavidin-HRP, and then TMB substrate was added for up to 20 min (Biolegend). After adding the TMB stop solution to terminate color development, relative absorbance was measured at 450 nm using a SpectraMax i3 microplate reader (Molecular Devices, San Jose, CA, USA).

### Coculture experiments and in vitro T cell killing assay

Murine lymphocytes and tumor-infiltrating CD8^+^ T cells (1 × 10^6^ cells/9.5 cm^2^) as effector cells were activated with Dynabeads T-Activator CD3/CD28 (Life Technologies, Carlsbad, CA, USA) for 72 h at 37 °C. Cells were stained with CellTrace™ Far Red Cell Proliferation Kit (Thermo Fisher Scientific, Waltham, MA, USA). The hPD-L1 MC38 cells (5 × 10^4^ cells/1.9 cm^2^) were treated with IFN-γ (10 ng/mL) for 24 h at 37 °C to induce PD-L1 reactive expression. The hPD-L1 MC38 cells were cocultured with the activated CD8^+^ T cells (2.5 × 10^5^ cells/1.9 cm^2^) at an effector cell-to-target cell ratio of 5:1 or with splenocytes (5 × 10^5^ cells/1.9 cm^2^) at an effector cell-to-target cell ratio of 10:1 and were treated with the indicated concentrations (1–4 μM) of Ce6, followed by PDT for 24 or 48 h at 37 °C.

### Statistical analysis

The data is presented as mean ± standard error of the mean. The mean difference between the treatment and control groups was taken into account when determining statistical significance using one-way ANOVA with Tukey's post hoc test for multiple comparisons. All statistical analyses were performed using GraphPad Prism software (v5.02; La Jolla, CA, USA). A *p*-value < 0.05 was considered statistically significant.

### Ethics approval and consent to participate

The study protocol and animal procedures were approved by the Animal Care and Use Committee of the Dongsung Cancer Center, Daegu, under the protocol IACUC #ds002205112-EUTO3.

## Supplementary Information

Supplementary Information.

## Data Availability

The data sets generated during and/or analyzed during the current study are available from the corresponding author upon reasonable request.

## Abbreviations

PDT: Photodynamic therapy
Ce6: Chlorine e6
PD-1: Programmed cell death-1
PD-L1: Programmed death-ligand 1
CD4: Cluster of differentiation
IL-2: Interleukin-2
DAMP: Danger-associated molecular pattern
ICD: Immunogenic cell death
APC: Antigen-presenting cells
ROS: Reactive oxygen species
APC: Antigen-presenting cells
NK: Natural killer cells
IFN-γ: Interferon-γ
NFAT: Nuclear factor of activated T cells,
