# Author Correction: Chlorin e6-associated photodynamic therapy enhances abscopal antitumor effects via inhibition of PD-1/PD-L1 immune checkpoint

**DOI:** 10.1038/s41598-023-35267-5

**Published:** 2023-06-01

**Authors:** Pallavi Gurung, Junmo Lim, Rajeev Shrestha, Yong‑Wan Kim

**Affiliations:** Dongsung Cancer Center, Dongsung Biopharmaceutical, Daegu, 41061 South Korea

Correction to: *Scientific Reports* 10.1038/s41598-023-30256-0, published online 21 March 2023

The original version of this Article contained errors in Figures 3 and 4, where the control groups were incorrectly incorporated into the graphs of each panel (A–E), respectively. The original Figures 3 and 4 and their accompanying legend appear below.

**Figure 3:**
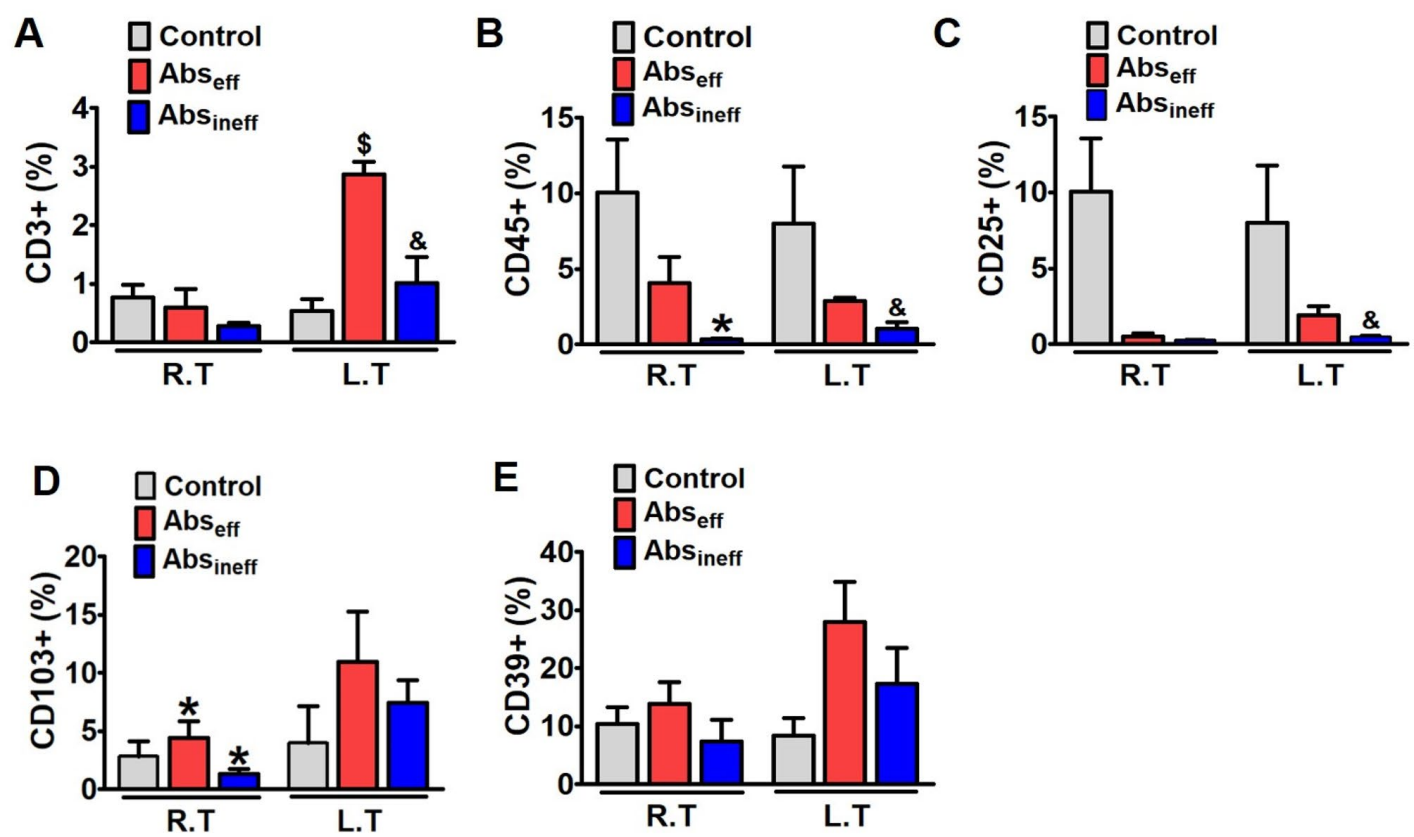
Enhanced accumulation and activation of T cells by Ce6-PDT in the melanoma mouse tumors. (**A**-**E**) Flow cytometry analysis to count and estimate the intratumoral fraction of (**A**) CD3^+^, (**B**) CD45^+^, (**C**) CD25^+^, (**D**) CD103^+^, and (**E**) CD39^+^ T cells, isolated from the irradiated right and non-irradiated left tumors in control, Abs_eff_ and Abs_ineff_ group. After 28 days of tumor cell injection, T cells in tumor tissues were isolated from B16F10 tumor-bearing mice. Data are from an experiment representative with n = 3 in the control, n = 3 in the abscopal effective, and n = 4 in the abscopal ineffective group. *P < 0.05 compared to right control tumor. **#P** < 0.05 compared to right tumor of the abscopal effective group, ^$^*P* < 0.05 compared to left control tumor, and ^&^*P* < 0.05 compared to left tumor of the abscopal effective group (by one-way ANOVA with Tukey’s post hoc test for multiple comparisons).

**Figure 4:**
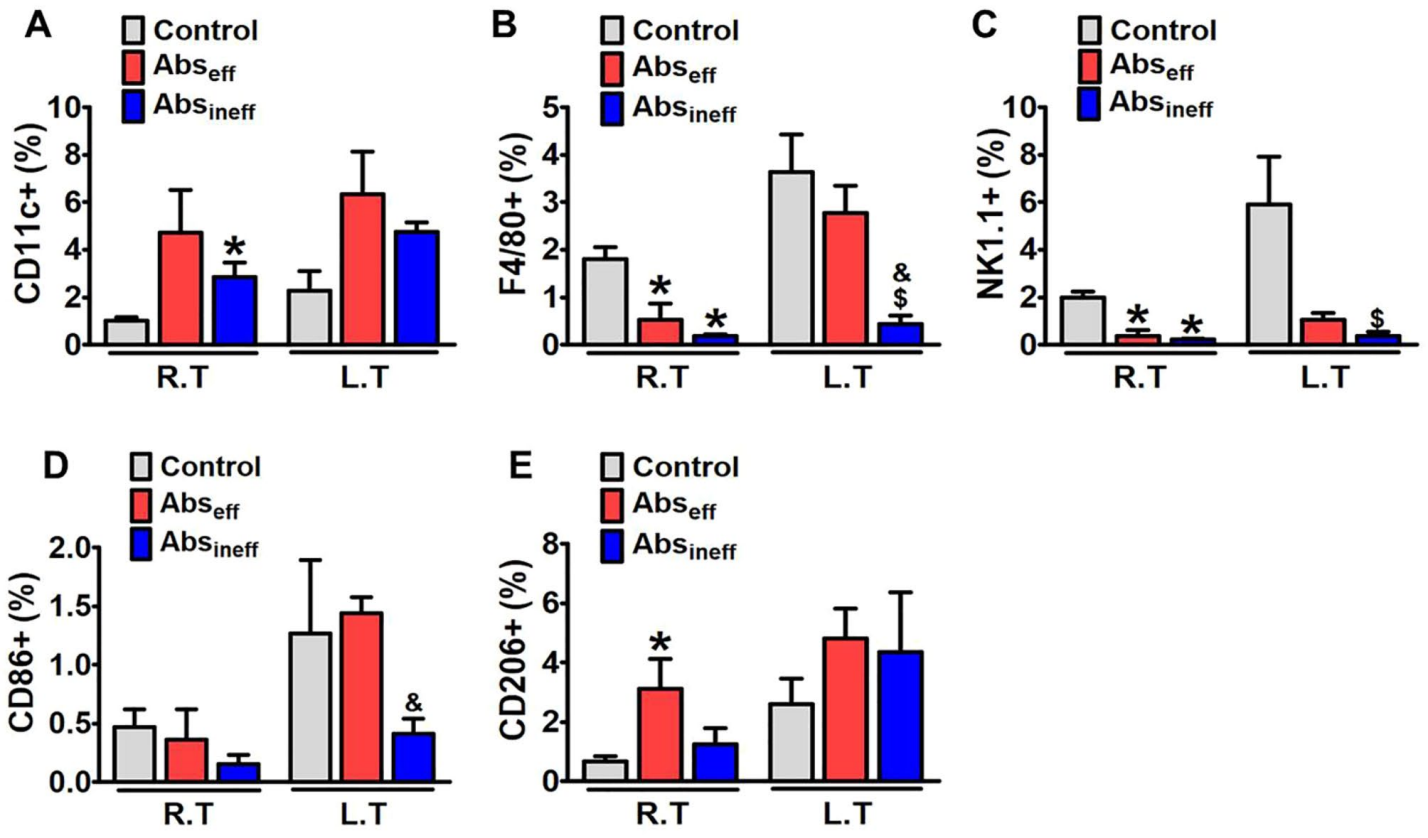
Flow cytometric analysis of the immune cell in the irradiated and non-irradiated tumor of melanoma mouse tumors**.** Percentages of (**A**) CD11c^+^, (**B**) F4/80^+^, (**C**) NK1.1^+^ (**D**) CD86^+^, and (**E**) CD 206^+^ in irradiated and non-irradiated tumor in control, Abs_eff_ group, and Abs_eff_ group. Data are from an experiment representative with n = 3 in the control, n = 3 in the effective, and n = 4 in the ineffective group. *P < 0.05 compared to right control tumor. **#P** < 0.05 compared to right tumor of the abscopal effective group, ^$^*P* < 0.05 compared to left control tumor, and ^&^*P* < 0.05 compared to left tumor of the abscopal effective group (by one-way ANOVA with Tukey's post hoc test for multiple comparisons).

As a result, the Figure legends of Figure 3 and Figure 4 contained errors, where for Figure 3

“*P < 0.05 compared to right control tumor. **#P** < 0.05 compared to right tumor of the abscopal effective group, ^$^*P* < 0.05 compared to left control tumor, and ^&^*P* < 0.05 compared to left tumor of the abscopal effective group (by one-way ANOVA with Tukey’s post hoc test for multiple comparisons).”

now reads:

“*p < 0.05 compared to irradiated right tumors in abscopal effective group. #*p* < 0.05 compared to irradiated right tumors in abscopal ineffective group (by one-way ANOVA with Tukey’s post hoc test for multiple comparisons).”

And for Figure 4,

“*P < 0.05 compared to right control tumor. **#P** < 0.05 compared to right tumor of the abscopal effective group, ^$^*P* < 0.05 compared to left control tumor, and ^&^*P* < 0.05 compared to left tumor of the abscopal effective group (by one-way ANOVA with Tukey’s post hoc test for multiple comparisons).”

now reads:

“*p < 0.05 compared to irradiated right tumors in abscopal effective group. #*p* < 0.05 compared to irradiated right tumors in abscopal ineffective group (by one-way ANOVA with Tukey’s post hoc test for multiple comparisons).”

The original Article has been corrected.

