# Supplementary material for: Chlorin e6-associated photodynamic therapy enhances abscopal antitumor effects via inhibition of PD-1/PD-L1 immune checkpoint
Source: Sci Rep. 2023 Mar 21;13:4647. doi: 10.1038/s41598-023-30256-0 (PMC10030802; doi:10.1038/s41598-023-30256-0)
Supplement: Supplementary file 1 — Supplementary Information. [file 41598_2023_30256_MOESM1_ESM.docx]

# Supplementary Information

**Chlorin e6-associated photodynamic therapy enhances abscopal antitumor effects via inhibition of PD-1/PD-L1 immune checkpoint**

Pallavi Gurung, Junmo Lim, Rajeev Shrestha and Yong-Wan Kim*

Dongsung Cancer Center, Dongsung Biopharmaceutical, Daegu 41061, South Korea

*Correspondence: [thomas06@hanmail.net](mailto:thomas06@hanmail.net) (Y-.W.K.).

**TABLE OF CONTENTS**

**S1. Splenic T cell distribution by Ce6-PDT–induced pancreatic cancer model.**

**S2. Splenic immune cell distribution by Ce6-PDT-induced pancreatic cancer mice**

**model.**

**S3. Gating strategy used in flow cytometry experiments.**

**S4. hPD-1 (Jurkat cells) and hPDL-1/TCR CHO-K1 cell viability following in vitro Ce6-**

**PDT.**

**S5. Data explaining the choice of dose (2.5 mg/kg) of Ce6 given to the mice.**

**S6. Data related to drug interval time before light irradiation in PDT, pharmacokinetics**

**or imaging study to determine the maximum amount of Ce6 in the tumor.**

**S7. Data explaining the effect of light alone on cell viability of B16F10 cells.**

**S1. Splenic T cell distribution in Ce6-PDT-induced pancreatic cancer mouse model.**

**
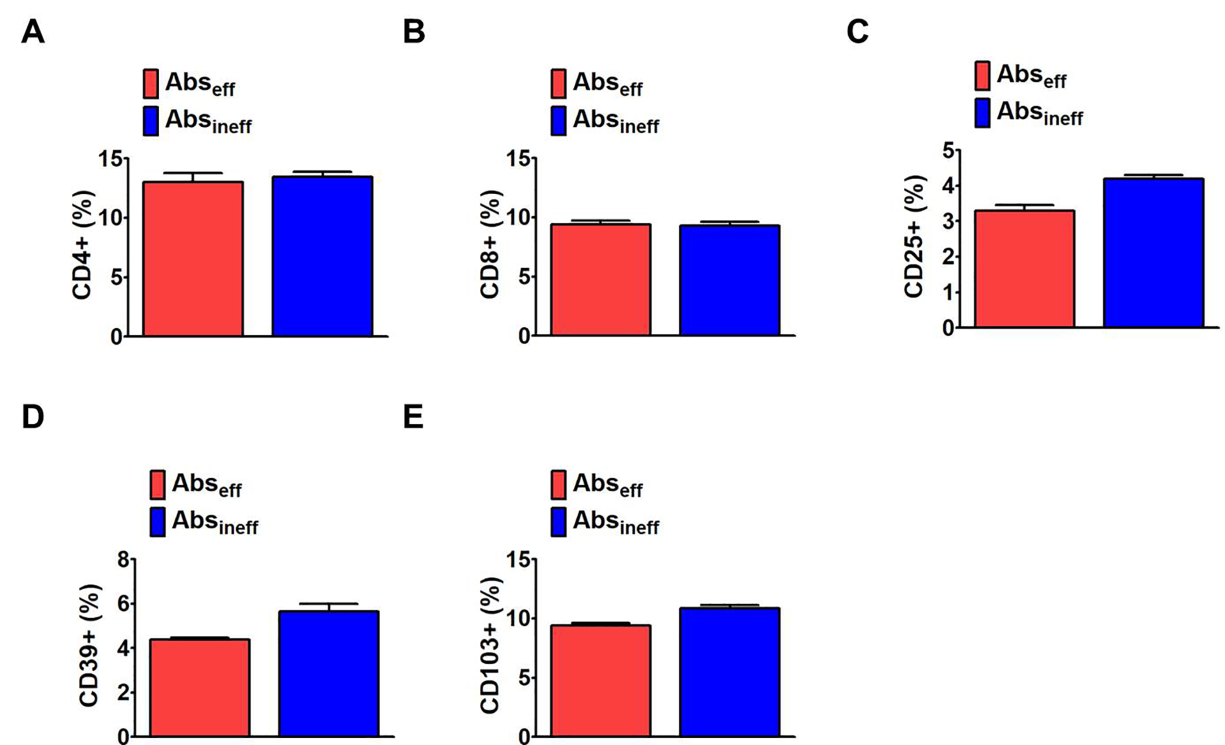
**

**Fig S1.** Ce6-PDT enhances the accumulation and activation of T cells in mice spleen of pancreatic cancer model. (A-E) Flow cytometry analysis to count and estimate the intratumoral fraction of (A) CD4+, (B) CD8+, (C) CD25+, (D) CD39+, and (E) CD103+ T cells isolated from spleen in abscopal effective (Abs_eff_ ) and abscopal ineffective (Abs_ineff_) group. After 28 days of tumor cell injection, T cells within the spleen were isolated from pancreatic tumor-bearing mice. Data are from an experiment representative of three mice in the effective and four mice in the ineffective group.

**S2. Splenic immune cell distribution by Ce6-PDT-induced pancreatic cancer mouse model.**

**
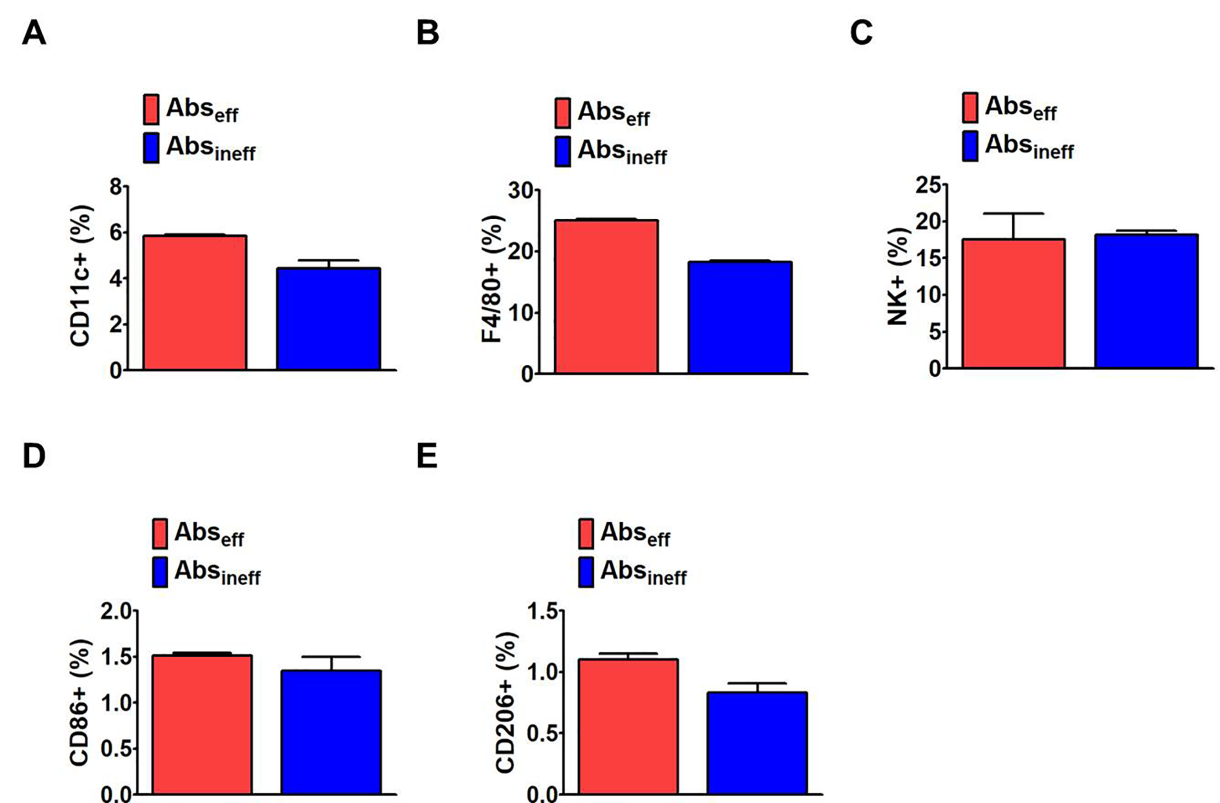
**

**Fig S2.** Ce6-PDT enhances the accumulation and activation of immune cells in mice spleen of pancreatic cancer model. (A-E) Flow cytometry analysis to count and estimate the intratumoral fraction of (A) CD11c+, (B) F4/80+, (C) NK+, (D) CD86+, and (E) CD206+  isolated from spleen in abscopal effective (Abs_eff_ ) and abscopal ineffective (Abs_ineff_) group. After 28 days of tumor cell injection, immune cell markers within the spleen were isolated from pancreatic tumor-bearing mice. Data from an experiment representative of three mice in the effective and four mice in the ineffective group.

**S3. Gating strategy used in flow cytometry experiments.**

**
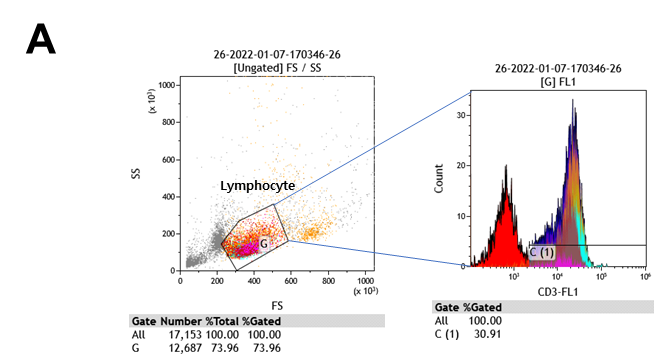
**

**
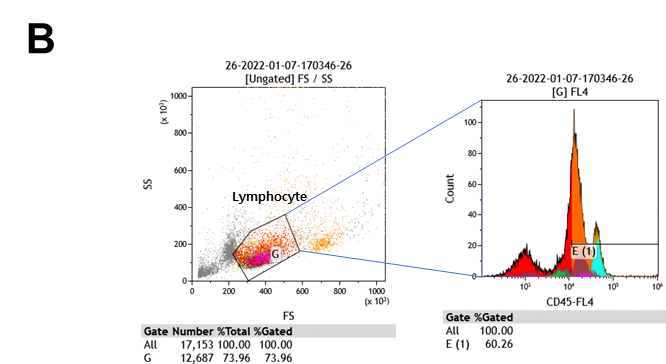
**

**
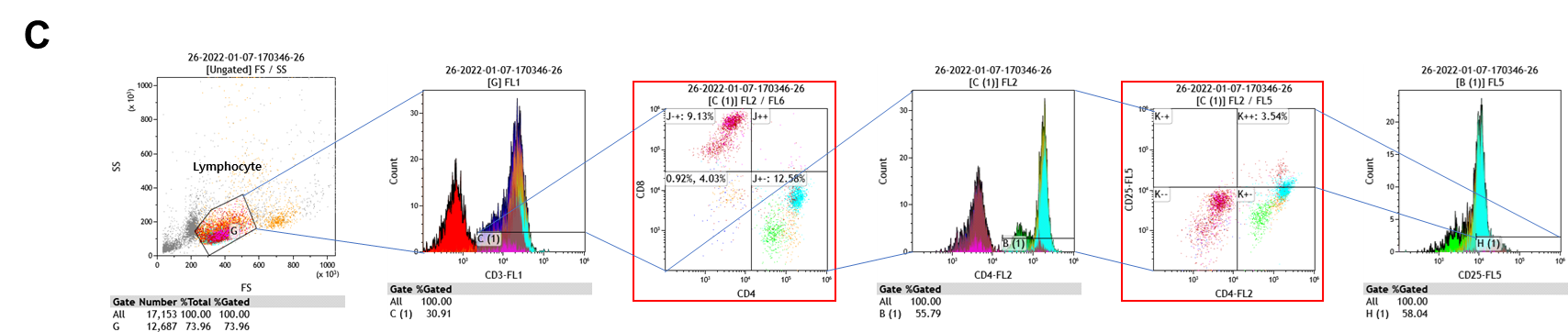
**

**
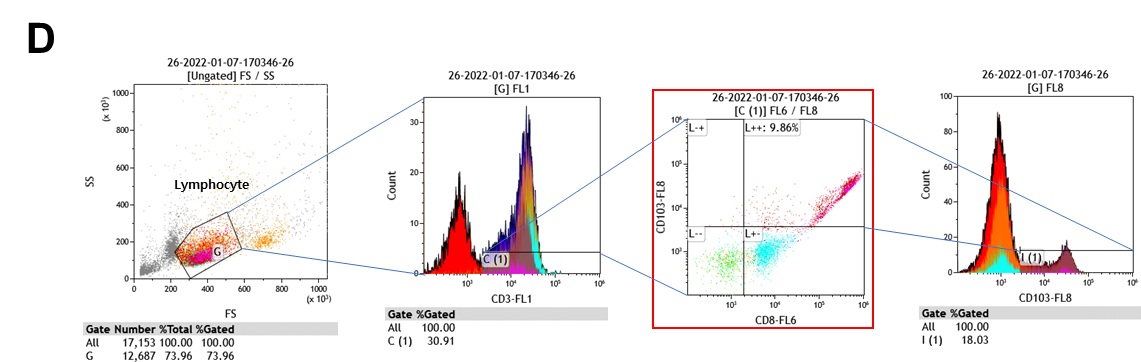
**

**
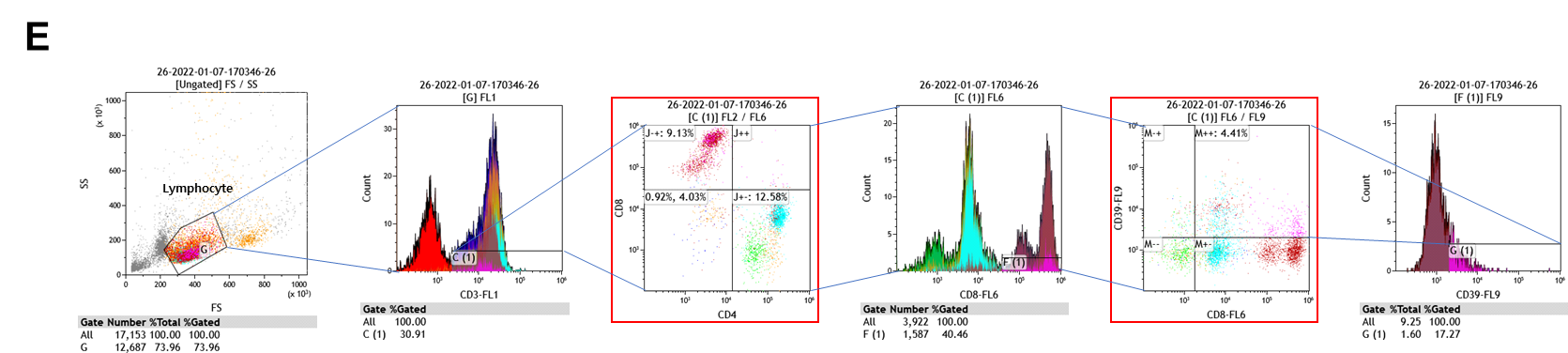
**

**Summary of filters and fluors**

| **FL1** | **FL2** | **FL4** | **FL5** | **FL6** | **FL8** | **FL9** |
| --- | --- | --- | --- | --- | --- | --- |
| CD3 | CD4 | CD45 | CD25 | CD8 | CD103 | CD39 |
| FITC | PE | PE Cy5.5 | PE Cy7 | APC | APC Cy7 | BV421 |

**Fig S3.** Flow cytometry gating strategy used to determine percentages of viable T cells and their subtypes. Lymphocytes were identified based on their characteristic properties in FSC versus SSC. (A) The subsets for CD3+T cells were identified. (B) The subsets for CD45+ T cells were identified. (C) The distribution of CD4+ and CD25+ in CD3+CD4+ T cells and a further subset of CD25+ T cells were identified. D) The distribution of CD8+ and CD103+ in CD3+ T cells and a further subset of CD103+ T cells were identified. E) The distribution of CD8+ and CD39+ in CD4+CD8+ T cells and a further subset of CD39+ T cells were identified.

**S4. hPD-1 (Jurkat cells) and hPDL-1/TCR CHO-K1 cell viability following *in vitro* Ce6-PDT.**


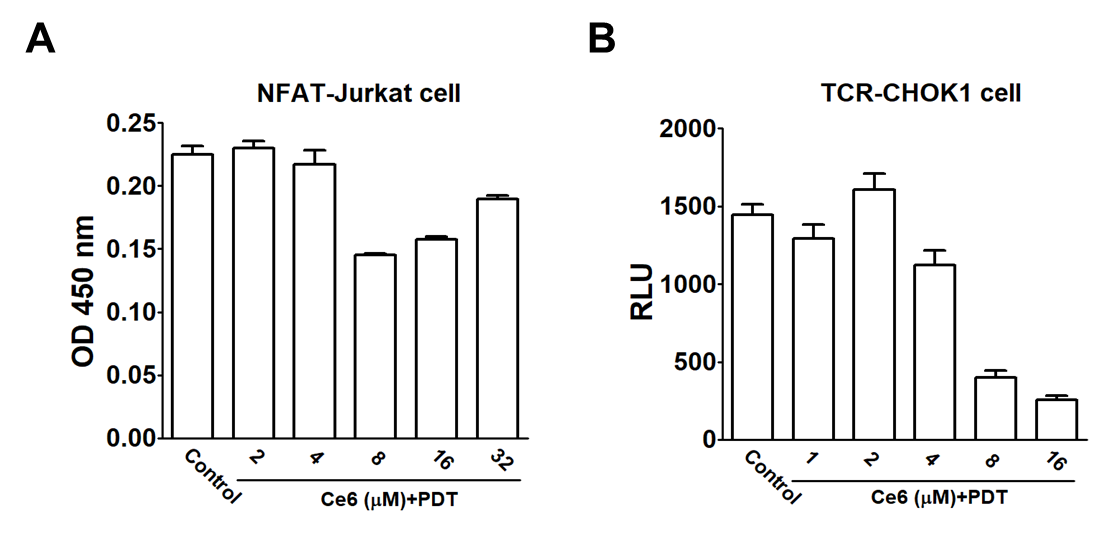


**Fig S4.** Cell viability of hPD-1 (Jurkat T cells) and hPD-L1/TCR CHO-K1 in 24 h following treatment with Ce6-PDT. (A) MTT assays were performed on hPD-1-Jurkat cells after Ce6 treatment (0 to 32 μM) for 3 h followed by irradiation with the light (660 nm, 50 mW, 5 J/cm^2^). (B) MTT assays were performed on hPD-L1/TCR CHO-K1 cells after Ce6 treatment (0 to 16 μM) for 3 h followed by irradiation with the light (660 nm, 50 mW, 5 J/cm^2^). Control cells were untreated cells.

**S5. Data explaining the choice of dose (2.5 mg/kg) of Ce6 given to the mice.**

**Table S5.1**: Dose-dependent effects of photosensitizers, Chlorin e6, Phonozen (PVP + Ce6) (1:1) with and without laser illumination on the extent and the rate of complete tumor necrosis of transplanted tumors (sarcoma M-1) in rats (n = 5 animals per subgroup; means ± SEM)

| **Treatment** | **Tumor volume**  **[cm^3^]** | **Extent of necrosis**  **[mm]** | **Rate of complete**  **necrosis**  **[%]** |
| --- | --- | --- | --- |
| Control | 1.11±0.25 | 0 | 0 |
| Chlorin e6: dose 2.5 mg/kg without illumination | 0.90±0.19 | 0 | 0 |
| Phonozen®: dose 2.5 mg/kg without illumination | 0.86±0.095 | 0 | 0 |
| Chlorin e6: dose 1 mg/kg + illumination at 50 J/cm^2^ | 0.64±0.036 | 6.4±0.75 | 20 |
| Phonozen®: dose 1 mg/kg + illumination at 50 J/cm^2^ | 0.85±0.15 | 8.8±0.86 | 80 |
| Chlorin e6: dose 2.5 mg/kg + illumination at 50 J/cm^2^ | 1.2±0.15 | 5.4±0.75 | 0 |
| Phonozen®: dose 2.5 mg/kg + illumination at 50 J/cm^2^ | 1.07±0.22 | 9.2±0.58* | 60 |
| Chlorin e6: dose 2.5 mg/kg + illumination at 100 J/cm^2^ | 0.66±0.047 | 8.8±0.37 | 40 |
| Phonozen®: dose 2.5 mg/kg + illumination at 100 J/cm^2^ | 0.96±0.087 | 11.6±0.51* | 100 |

*P<0.05 compared to a corresponding group of rats receiving Chlorin e6 (Student´s t-test)

**S5.1. Correlations with in vitro assays regarding 2.5 mg/kg Ce6 given in *in vivo* study.**


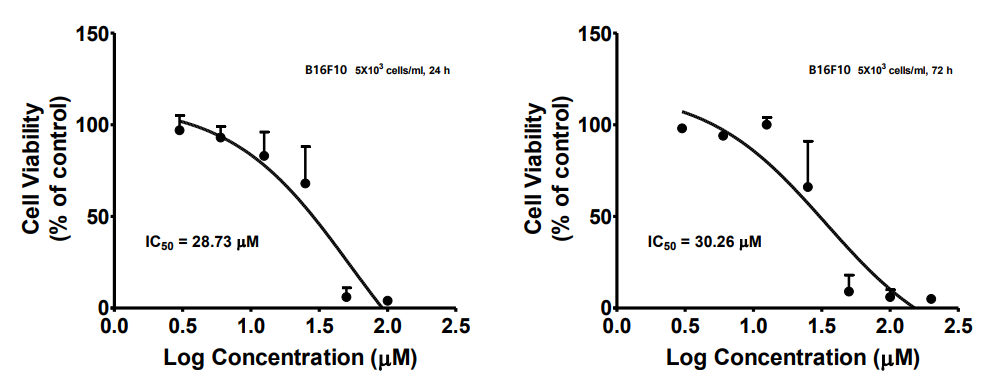


**Fig S5.1** Cell viability of B16F10 cells in 24 h and 72 h against Ce6.

We have shown IC50 of B16F10 cells. But it is not easy to make in vitro dose correlations with *in vivo* dose. Instead, we have shown 10-day repeated intravenous dose toxicity data at doses of 25, 50, and 100 mg/kg once daily.

**S.5.2 Experimental description:**

Total mice: 115 (Male:57) and (Female:57)

Total mice used for the experiment: 104 (Male:52) and (Female:52)

Age of mice when received: 6 weeks

Age of mice during the injection: 7 weeks

Body weight of mice during the injection: 30.3-36.8 g (Male), 23.8-28.7 g (Female)

Body weight of only male ICR mice were used for Tumor inhibition assay.

**Table S5.2: 10-day repeated intravenous dose toxicity study of Phonozen in ICR mice.**

| **Group** | **Sex** | **No. of Animals** | **Animal ID** | | **Volume**  **(mL/kg)** | **Dose**  **(mg/kg)** |
| --- | --- | --- | --- | --- | --- | --- |
|  |  |  | **Dosing** | **Recovery** |  |  |
| **Vehicle control**  **(VC)** | Male | 16 | 1-10 | 11-16 | 10 | 0 |
|  | Female | 16 | 53-62 | 63-68 |  |  |
| **(Test 1) T1** | Male | 10 | 17-26 | - | 10 | 25 |
|  | Female | 10 | 69-78 |  |  |  |
| **(Test 2) T2** | Male | 10 | 27-36 | - | 10 | 50 |
|  | Female | 10 | 79-88 |  |  |  |
| **(Test 3) T3** | Male | 16 | 37-46 | 47-52 | 10 | 100 |
|  | Female | 16 | 89-98 | 99-104 |  |  |

| **Males** | | | | Unit: g |
| --- | --- | --- | --- | --- |
| **Treatment** | | | | |
|  | | | | |
| **Group** |  | **Day: 1** | **Day: 8** | **Day: 10** |
| **#** |  | **Session: 1** | **Session: 1** | **Session: 1** |
| **VC** | **(n)** | 16 | 16 | 16 |
|  | **Means** | 32.5 | 35.0 | 35.5 |
|  | **S.D** | 1.38 | 1.65 | 1.80 |
|  |  |  |  |  |
| **T1** | **(n)** | 10 | 10 | 10 |
|  | **Means** | 32.7 | 34.4 | 34.6 |
|  | **S.D** | 0.68 | 1.16 | 1.33 |
|  |  |  |  |  |
| **T2** | **(n)** | 10 | 10 | 10 |
|  | **Means** | 32.9 | 33.9 | 34.5 |
|  | **S.D** | 1.57 | 1.70 | 1.55 |
|  |  |  |  |  |
| **T3** | **(n)** | 16 | 16 | 16 |
|  | **Means** | 32.3 | 33.4+D | 34.3 |
|  | **S.D** | 1.58 | 1.35 | 1.44 |

**Table S5.3: Summary of body weight of male ICR mice with different treatment sessions.**

**Mouse/CrljOri:CD1 Repeat Dose Toxicity/Tox**

**+D = Dunnett LSD Test Significant at the 0.01 level**

**Fig S5.2**: Body weight values of ICR male mice intravenously administered with Phonozen. Data are expressed as mean ± SD.

**S6.** **Data related to drug interval time before light irradiation in PDT, pharmacokinetics or imaging study to determine the maximum amount of Ce6 in the tumor.**

**Fig S6**: Enrichment of Phonozen and Ce6 in the tumor (M-1 sarcoma) and in normal tissue of the femur at different time intervals after intravenous administration at 1 mg/kg (rats, n = 3 animals per measurement point).

**S7. Data explaining the effect of light alone on cell viability of B16F10 cells.**

**
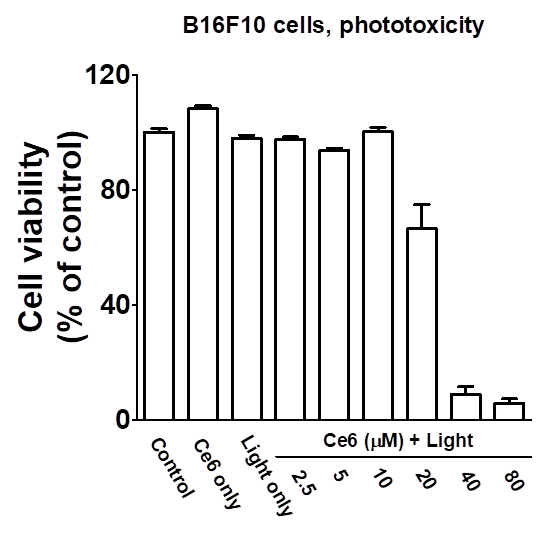
**

**Fig S7.** *In vitro* PDT of cancer cells. Cell viability of the B16F10 cells that were incubated with or without various concentrations of free Ce6 (2.5-80 μΜ) for 3 h followed by irradiation with 660 nm, (50 mW, 5 J/cm^2^).  Cell viability in the control, Ce6-treated, and laser only-treated groups remain unchanged.
